# Profiling of phytohormone‐specific microRNAs and characterization of the miR160‐ARF1 module involved in glandular trichome development and artemisinin biosynthesis in *Artemisia annua*

**DOI:** 10.1111/pbi.13974

**Published:** 2022-12-20

**Authors:** Zhiying Guo, Kai Hao, Zongyou Lv, Luyao Yu, Qitao Bu, Junze Ren, Henan Zhang, Ruibing Chen, Lei Zhang

**Affiliations:** ^1^ Medical School of Nantong University Nantong China; ^2^ School of Food and Bioengineering Fujian Polytechnic Normal University Fuqing China; ^3^ Department of Pharmaceutical Botany School of Pharmacy, Naval Medical University Shanghai China; ^4^ Research and Development Center of Chinese Medicine Resources and Biotechnology Shanghai University of Traditional Chinese Medicine Shanghai China; ^5^ Institute of Edible Fungi, Shanghai Academy of Agricultural Sciences, National Engineering Research Center of Edible Fungi Shanghai China; ^6^ Key Laboratory of Edible Fungi Resources and Utilization (South), Ministry of Agriculture Shanghai China; ^7^ Innovative Drug R&D Center, College of Life Sciences Huaibei Normal University Huaibei China

**Keywords:** *Artemisia annua*, artemisinin, phytohormone, glandular trichome, miR160‐ARF1

## Abstract

MicroRNAs (miRNAs) play crucial roles in plant development and secondary metabolism through different modes of sequence‐specific interaction with their targets. Artemisinin biosynthesis is extensively regulated by phytohormones. However, the function of phytohormone‐responsive miRNAs in artemisinin biosynthesis remains enigmatic. Thus, we combined the analysis of transcriptomics, small RNAs, and the degradome to generate a comprehensive resource for identifying key miRNA‐target circuits involved in the phytohormone‐induced process of artemisinin biosynthesis in *Artemisia annua*. In total, 151 conserved and 52 novel miRNAs and their 4132 targets were determined. Based on the differential expression analysis, miR160 was selected as a potential miRNA involved in artemisinin synthesis. Overexpressing *MIR160* significantly impaired glandular trichome formation and suppressed artemisinin biosynthesis in *A. annua*, while repressing its expression resulted in the opposite effect, indicating that miR160 negatively regulates glandular trichome development and artemisinin biosynthesis. RNA ligase‐mediated 5′ RACE and transient transformation assays showed that miR160 mediates the RNA cleavage of *Auxin Response Factor 1* (*ARF1*) in *A. annua*. Furthermore, ARF1 was shown to increase artemisinin synthesis by activating *AaDBR2* expression. Taken together, our results reveal the intrinsic link between the miR160‐ARF1 module and artemisinin biosynthesis, and may expedite the innovation of metabolic engineering approaches for high and stable production of artemisinin in the future.

## Graphical abstract

miR160 responses to SA, JA induction and forms a module with target gene *ARF1* to regulate glandular trichome development and artemisnin biosynthesis in *Artemisia annua*.

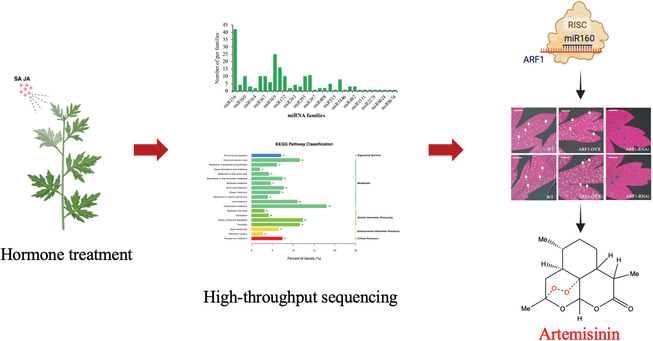

## Introduction

Malaria is a global health problem with 241 million cases in 87 endemic countries in 2020 (WHO, 2021). *Artemisia annua* L. has gained increasing attention for its widespread use in the extraction of a potent drug for malaria, artemisinin. Artemisinin is a sesquiterpenoid produced in glandular trichomes of *A. annua* (Singh *et al*., 2016). The oral delivery of artemisinin in the form of dried *A. annua* leaves has proven highly effective even against parasite strains resistant to artemisinin combination therapy and intravenous artesunate (He *et al*., 2021). In addition to antimalarial benefits, artemisinin has many other biological and pharmacological properties, including antiviral, anticancer, and antischistosomal effects (Shen *et al*., 2016b). Therefore, artemisinin has been considered to be a promising multifunctional natural product. The relatively low content (0.1%–1.0% of dry weight) of artemisinin in *A. annua* is a serious limitation to the commercialization of the drug (Shen *et al*., 2018). Although semisynthesis of artemisinin via artemisinic acid can be obtained from genetically modified yeast, the semisynthetic production of artemisinin is expensive and thus cannot replace its agricultural production at present (Paddon *et al*., 2013). Hence, regulating artemisinin biosynthesis in *A. annua* to increase its content remains the desirable approach to resolve the contradiction between supply and demand.

Concerted attempts have been made to elucidate the biosynthetic pathway of artemisinin and its regulatory mechanisms in *A. annua* (Olofsson *et al*., 2011; Tan *et al*., 2015; Zhou *et al*., 2020). In the last three decades, three primary metabolic engineering strategies have been developed to optimize the production of artemisinin in *A. annua*: (i) overexpressing artemisinin biosynthetic pathway genes (*ADS*, *CYP71AV1*, *DBR2*, and *ALDH1*); (ii) overexpressing transcription factors (TFs) involved in artemisinin biosynthesis and glandular trichome formation; and (iii) applying exogenous phytohormones (Olofsson *et al*., 2011; Pulice *et al*., 2016; Shen *et al*., 2016b). Methyl jasmonate (MeJA), salicylic acid (SA), abscisic acid (ABA), and gibberellins (GAs) are effective elicitors for enhancing artemisinin accumulation by inducing the expression of genes encoding TFs that regulate the artemisinin biosynthetic pathway genes or increase the glandular trichome density in *A. annua* (Chen *et al*., 2021; Kumari *et al*., 2018; Ma *et al*., 2018; Zhang *et al*., 2013). *Aa*MYC2, a JA‐response bHLH TF, can bind to the G‐box‐like cis‐elements present in both *CYP71AV1* and *DBR2* promoters and then strongly activate their expression (Shen *et al*., 2016a). *Aa*TCP15, a JA and ABA dual‐responsive teosinte branched1/cycloidea/proliferating (TCP) TF, is essential for JA‐ and ABA‐induced artemisinin biosynthesis and functions by directly binding to and activating the promoters of *DBR2* and *ALDH1*, two genes encoding enzymes for artemisinin biosynthesis (Ma *et al*., 2021). Glandular trichome initiation occurs at the G1 to S and G2 to M stages, and MeJA may specifically delay the switch from G1 to S and prolong the G1 phase (Noir *et al*., 2013; Schnittger *et al*., 2002). Consequently, exogenous treatment with JA stimulates artemisinin production in *A. annua*, as well as the formation of glandular trichomes (Baldi and Dixit, 2008). Similarly, the external application of ABA can activate the TF *Aa*bZIP1, directly regulating the accumulation of artemisinin by stimulating the expression of *ADS*, *FPS*, and *CYP71AV1* (Zhang *et al*., 2013). Some studies have revealed that SA applications are able to increase artemisinin content by 54% in two different ways: converting the dihydroartemisinic acid into artemisinin due to the burst of ROS and positively affecting the expression of artemisinin‐related biosynthetic enzymes (Pu *et al*., 2009). In addition, the expression of some enzyme genes such as the *β* glucosidase gene, to increase glandular trichome density can improve artemisinin content (Singh *et al*., 2016).

In addition to the TF, increasing evidence suggests that the miRNA‐TF module has emerged as a key regulator of phytohormone response pathways in planta by affecting their metabolism (Lv *et al*., 2017). For instance, the phytohormone‐responsive miR156‐SPL module is involved in phase changes, leaf trichome development, anthocyanin biosynthesis, and plant responses to salt stress in *Arabidopsis thaliana* (Gou *et al*., 2011; Yu *et al*., 2010). Similarly, the miR156‐SPL module has been proven to function in regulating developmental phase transition and flowering and in the spatiotemporal regulation of sesquiterpene biosynthesis. For instance, miR156 plays a role in regulating the formation of (*E*)‐*β*‐caryophyllene in the flowering stage by targeting SPL9, which is a positive regulator of *TPS21* in sesquiterpene biosynthesis (Yu *et al*., 2015). These studies implicate the possibility that miRNAs are involved in artemisinin synthesis. Thus, further systematic characterization of the miRNA is still needed to reveal their regulatory mechanisms in artemisinin biosynthesis.

Although computational predictions and high‐throughput sequencing of miRNAs have been performed previously for *A. annua*, the miRNAs related to artemisinin synthesis cannot be accurately screened in the absence of reference genomes (Khan *et al*., 2020; Pani *et al*., 2011; Perez‐Quintero *et al*., 2012). Herein, in view of the diverse regulatory effects of phytohormones on artemisinin synthesis, a pipeline to identify candidate miRNAs under various hormone stress conditions was designed to decipher the regulatory roles of miRNAs in artemisinin biosynthesis in *A. annua* at the genome‐wide level. Sequencing and bioinformatic methods were used for the first time to profile miRNAs in *A. annua* with ABA, MeJA, and SA treatment. Furthermore, the phytohormone‐responsive miR160‐ARF1 module was identified as a negative regulator in repressing glandular trichome development and artemisinin biosynthesis in *A. annua*. Collectively, these findings provide not only new insights into the important roles of the miRNA‐TF module in the development of glandular trichomes and the regulation of artemisinin biosynthesis but also a new strategy to engineer plants for high and stable production of artemisinin in the future.

## Results

### Profiling of miRNAs in *A. annua*

**Figure 1:**
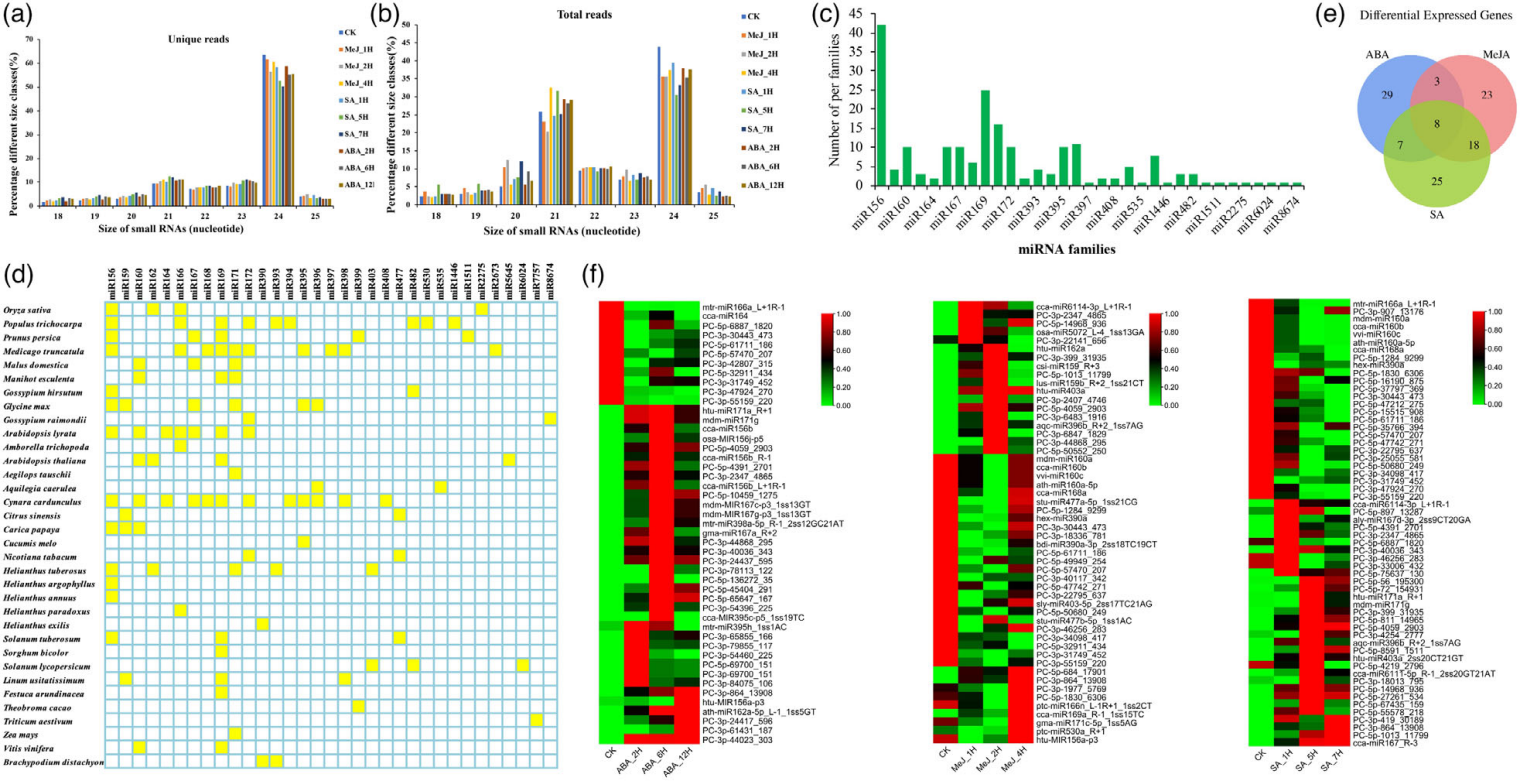
miRNA features of *Artemisia annua*. (a) Length distribution of unique sRNAs in *A. annua* sequencing samples. (b) Length distribution of total sRNAs in *A. annua* sequencing samples. (c) Number of conserved miRNA families from *A. annua*. (d) Conserved miRNA families in *A. annua* and across species. (e) Venn diagram showing the overlap of significantly differentially expressed miRNAs between the control and ABA‐, SA‐, and MeJA‐treated in *A. annua* groups. (f) Differentially expressed miRNA analysis. Heatmaps show the significantly differential expression of miRNAs (*P* < 0.05) under ABA, MeJA, and SA treatment in *A. annua*. Red indicates higher levels of miRNA and green indicates lower levels. The absolute signal intensity ranges from 0 to +1.0, with corresponding colour changes from green to red.

To study the post‐transcriptional regulation, regarding especially miRNAs involved in the molecular regulation of artemisinin biosynthesis, 10 small RNA (sRNA) libraries were constructed from aerial tissue of *A. annua* treated with DMSO and MeJA, SA and ABA at different time points. The numbers of raw reads and valid reads, which were obtained from one control and nine treatments, are summarized in Table S1. The size distributions of the unique valid sRNAs (Figure 1a) and total valid sRNAs (Figure 1b) in the 10 libraries showed strikingly similar patterns. The valid reads ranging from 20 to 24 nucleotides were approximately 89% and 90% in the total and unique sequences, respectively, with 21‐ and 24‐nt reads being the most dominant sRNA species, consistent with evidence found in the dicotyledonous plants (Guo *et al*., 2017).

To identify the conserved miRNAs from 10 *A. annua* libraries, all valid reads were mapped onto the genome of *A. annua* and the sequences of known plant miRNAs, such as miRNA precursors and mature miRNAs registered in miRbase 21.0. The sequences mapped to miRBase were identified as conserved miRNA, and those mapped to the *A. annua* genome only were considered novel miRNAs. The criteria of the blast search required no more than two mismatches in the first 16 nt of the miRNA and three mismatches in total between the specific miRNAs and pre‐miRNAs in miRBase. All detected miRNAs were categorized into Groups 1–4 (gp1‐4), and members of gp4 were identified as novel miRNA candidates. Interestingly, the numbers of miRNAs varied among families. The miR156 family was the largest (42 members), followed by the miR169 family (25 members), and more than 1/3 of miRNA families had only one miRNA member (Figure 1c). Following the BLASTn search and further sequence analysis, 224 conserved miRNAs belonging to 33 families in the small RNA library were found to be orthologues of known miRNAs from other plant species, which were previously deposited in the miRBase database (Figure 1d). The distribution of the conserved miRNA family in *A. annua* was highly similar to those of *Artichoke*, *Jerusalem artichoke*, and other species belonging to Compositae, and was significantly different from those of other species. In total, 61 pre‐miRNAs, corresponding to 52 mature miRNAs, were identified as novel miRNA candidates that were not registered in miRBase. The number of miRNAs was counted and normalized to the total reads of sRNAs. The expression levels of miRNA families of miR156, miR166, miR167, miR393, miR171, and miR160 were high in our pooled *A. annua* sample. Precursors forming hairpin structures for the novel miRNAs were predicted, with folding free energies ranging from −157.8 to −35.5 kcal/mol (Table S2). The lengths of precursors of the novel miRNAs ranged from 81 nt (PC‐5p‐1705_6778) to 252 nt (PC‐5p‐1284_9299).

### Response patterns of miRNAs to phytohormones

To systematically identify miRNAs in *A. annua* that respond to phytohormones and may be involved in the biosynthesis of artemisinin, the differential expression of miRNAs in 10 libraries was analysed and compared based on the normalized read counts generated from the high‐throughput sequencing. Generally, the majority of miRNAs (77, 68.0% of the differentially expressed miRNAs) were upregulated in hormone‐treated samples compared with their levels in the control. There were 23 MeJA‐specific miRNAs, of which 13 miRNAs were conserved and 10 miRNAs were novel. Four conserved miRNAs and 21 novel miRNAs were specific to SA treatment. Meanwhile, 12 conserved miRNAs and 17 novel miRNAs were specific to ABA treatment. Additionally, one conserved miRNA and 2 novel miRNAs were found in the ABA and MeJA treatments but not in the SA treatment. Similarly, 3 conserved miRNAs and 4 novel miRNAs were detected only in the ABA and SA treatments, whereas 8 conserved miRNAs and 10 novel miRNAs were detected only in the MeJA and SA treatments. In comparing the three treated groups with each other, only 8 novel miRNAs were all significantly differentially expressed, but the expression levels were low (Figure 1e). In total, 41 known miRNAs and 72 novel miRNAs were found to be differentially expressed in at least two pairwise comparisons when stricter criteria of total expression abundance >10 and *P*‐value ≤0.05 were used in hormone‐treated groups compared with the mock treatment group. The heatmap of potential differentially expressed miRNAs is illustrated in Figure 1f.

**Figure 2:**
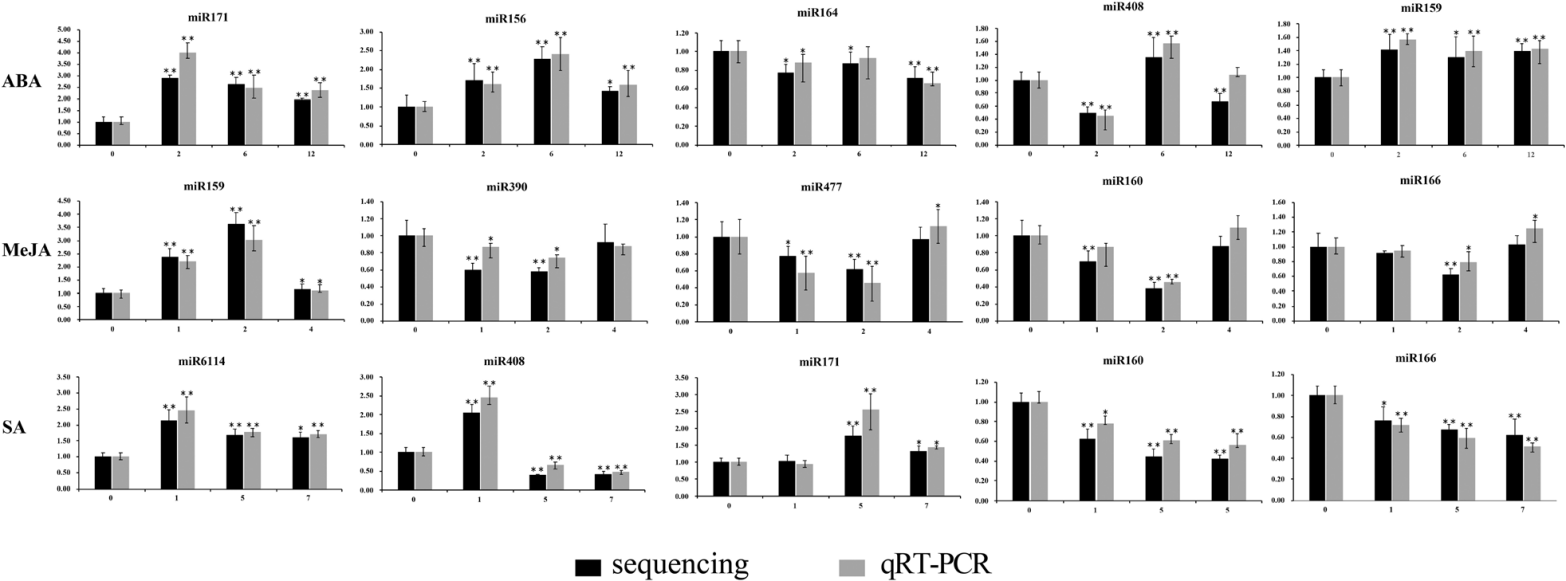
Confirmation of the expression levels of selected miRNAs in *Artemisia annua* by qRT‐PCR. The horizontal axis represents the time after hormone treatments (hours), and the vertical axis represents the relative expression of miRNAs. The black bar represents the deep sequencing, and the grey bar represents the qRT‐PCR. The expression levels of these miRNA were confirmed using stem‐loop qRT‐PCR. *Actin* was used as a loading control in qRT‐PCR. The data are represented as the mean plus SD of *n* = 3 biological replicates. *P < 0.05, **P < 0.01, Student's *t* test.

qRT‐PCR was used to investigate the miRNA expression profiles of the selected miRNAs. As shown in Figure 2, the sequencing and the qRT‐PCR results showed that miR156 expression first increased and then decreased with ABA treatment. Conversely, the miR160 showed a trend of first decreasing and then increasing again with MeJA treatment, but miR160 showed a continuous decreasing trend after SA treatment. miR171 was significantly differentially expressed by ABA or SA induction, and miR159 was significantly differentially expressed by ABA or MeJA induction. In comparing results from qPCR and sequencing analyses, the expression levels of these miRNAs between qPCR and sequencing analyses were consistent (Figure 2). Therefore, three conserved miRNAs (miR159, miR160, and miR171), which respond to the induction of two hormones, were used as candidates for artemisinin biosynthesis.

### Identification of miRNA target genes in *A. annua* by degradome sequencing

Multiple studies have indicated that miRNAs may directly target TFs that affect plant development and various secondary metabolism processes (Samad *et al*., 2017). Degradome sequencing combines the advantages of high‐throughput deep sequencing, computer analysis, and RACE to search for miRNA‐guided cleaved sites in mRNAs and was used as an efficient strategy to globally identify small RNA targets in this study. Through degradome sequencing, among the 224 miRNAs of the 33 known miRNA families, 190 could be searched for their potential targets, yielding 254 genes (Table S3). Among the 345 novel miRNAs, there were 328 predicted targets (Table S3). The detailed annotation of each miRNA target is shown in Table S3. One‐half of the conserved miRNA targets (131 of 258) were TFs, including SPL, GRAS, AP2, ARF, MYB, and NAC. Other conserved miRNA targets, such as CYP85A1, COMT1, and GA2OX1, are involved in secondary metabolism.

According to Gene Ontology (GO) annotation classification, the category containing the most target genes was ‘biological process’, which was subcategorized into the 25 biological processes displayed in Figure S1, and regulation of transcription process (GO: 0006355), oxidation–reduction process (GO: 0055114), and auxin‐activated signalling pathway (GO: 0009734) were the most significantly enriched terms (Figure S1). Kyoto Encyclopedia of Genes and Genomes (KEGG) enrichment analysis of 72 miRNAs/miRNA families and 184 target genes revealed that plant hormone signal transduction (ko04075), monoterpenoid biosynthesis (ko00902), plant‐pathogen interaction (ko04626), and purine metabolism (ko00230) pathways were the most important pathways (Figure S2). Therefore, the focus of the further study was on miRNAs in the plant hormone signal transduction category.

In the present study, we also found expression changes for several miRNAs involved in the regulation of transcription and auxin signalling. For instance, miR160 showed a 3.78‐fold change in abundance under SA or MeJA treatment (Figures 1f, 2), highlighting it as a candidate for having a regulatory role in artemisinin synthesis. miR160 was predicted to target an Auxin_resp domain TF, which functions as a hormone‐responsive TF. ARF TFs are also regulated by miR160 and function in plant growth and the response to stress in *A. thaliana* (Liu *et al*., 2016). Based on the above, an attempt was made to identify the function of miR160 in *A. annua*.

### miR160 regulates artemisinin biosynthesis and glandular trichome development

**Figure 3:**
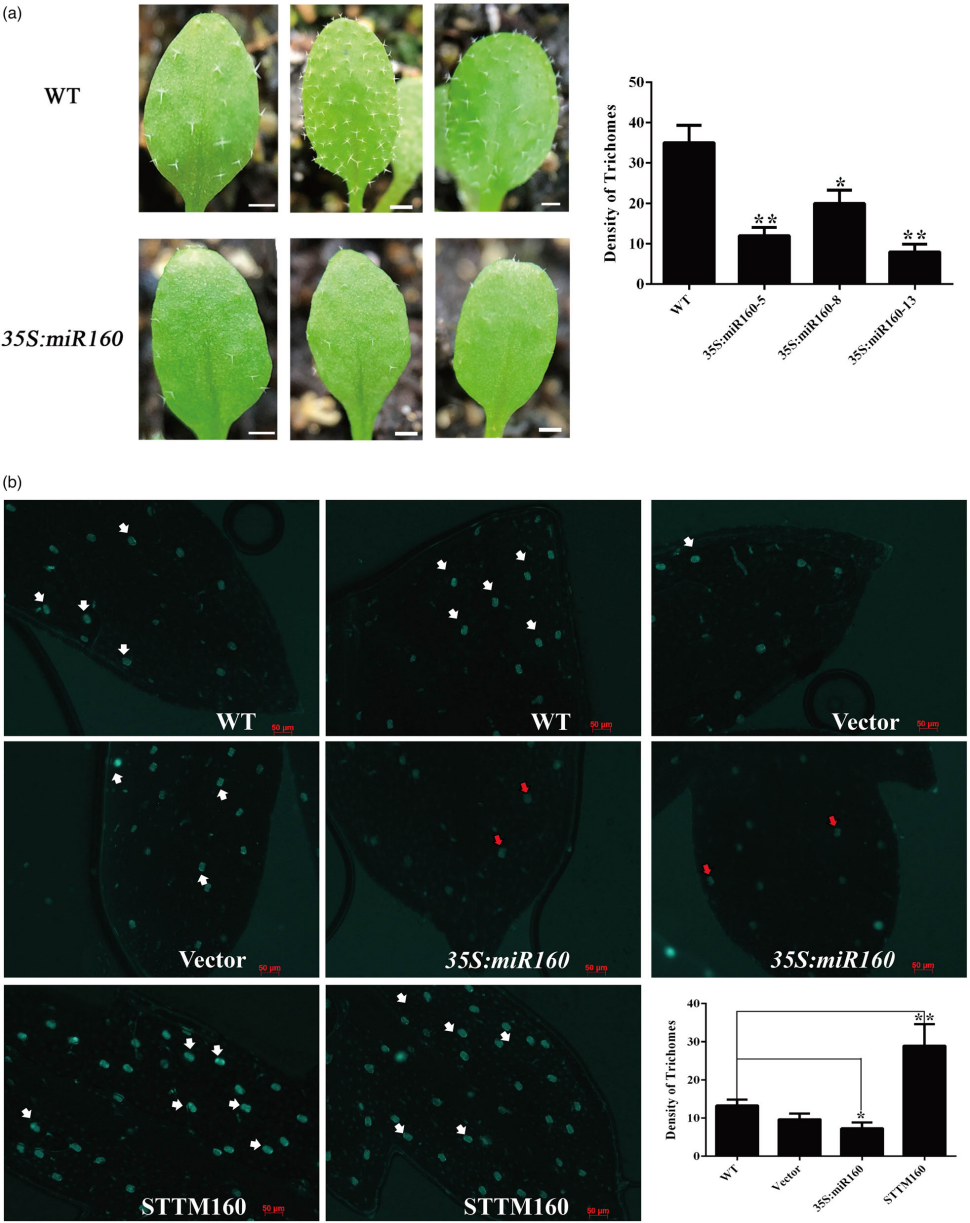
Density of trichomes in leaves of transgenic *Arabidopsis thaliana* and *Artemisia annua* plants. (a) Leaf surface and density of trichomes in leaves from WT plants and *35S:miR160* transgenic *A. thaliana* plants. The number of trichomes decreased significantly compared with WT. Bars represent 1 mm. *P < 0.05, **P < 0.01, Student's *t* test. (b) The adaxial surface was observed using fluorescence microscopy of leaves and the density of glandular trichomes in leaves from *35S:miR160* and STTM160 transgenic *A. annua* lines, plants transformed with the empty vector, and WT plants. Bars represent 50 μm. *P < 0.05, **P < 0.01, Student's *t* test.

Since some of the predicted or validated ARFs targeted by miR160 are orthologous to the tomato ARFs involved in the formation of trichomes (Zhang *et al*., 2015), we theorized that miR160 might also be involved in glandular trichome development in *A. annua*. To test this hypothesis, *MIR160* overexpression vectors were transformed into the *Agrobacterium strain* GV3101, which was then introduced into *A. thaliana* Col‐0 by the *Agrobacterium*‐mediated floral dip method. Three candidate *MIR160* overexpression lines (named *35S:miR160*), *35S:miR160*‐5, 8, and 13, were chosen for further analysis. For each transgenic line, three 2‐week‐old third leaves were used to count the number of trichomes. For comparison, a significantly decreased trichome density was found on the first two rosette leaves in *35S:miR160* transgenic lines, with a 43–77% decrease compared to wild‐type (WT) plants (Figure 3a).

**Figure 4:**
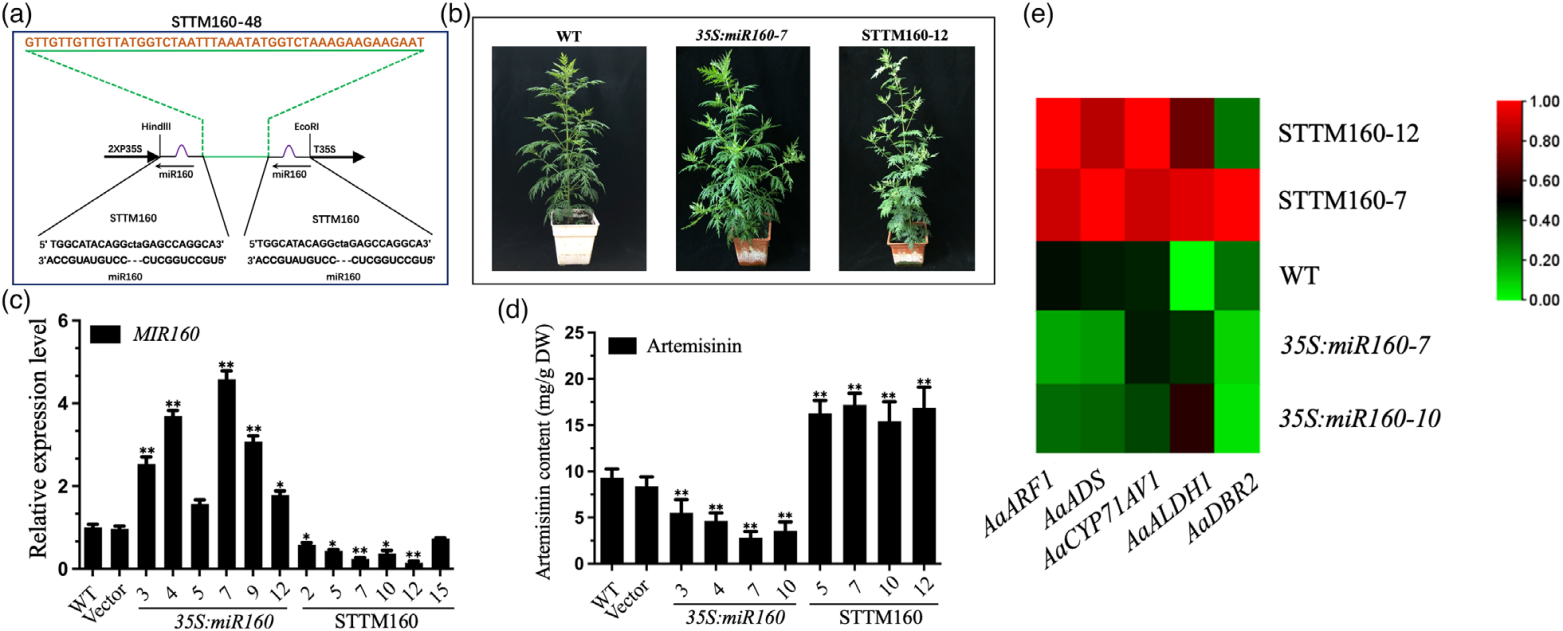
Analysis of miR160 transgenic *Artemisia annua* plants. (a) Schematic representation of STTM constructs used for silencing miR160 via *Agrobacterium tumefaciens*‐mediated transient expression. Green indicates the spacer region and the spacer sequence. Purple indicates the bulge sequences in the miRNA‐binding sites. (b) Different phenotypes among the *35S:miR160*, STTM160, and WT lines. (c) Expression levels of *MIR160* in the different *35S:miR160* and STTM160 lines, plants transformed with the empty vector, and WT plants. *Actin* was used as the internal standard. All data represent the means ± SDs of three replicates from three cutting propagations. *P < 0.05, **P < 0.01, Student's *t* test. (d) HPLC analysis of artemisinin in the leaves of different *35S:miR160* and STTM160 lines, plants transformed with the empty vector, and WT plants. All data represent the means ± SDs of three replicates from three cutting propagations. *P < 0.05, **P < 0.01, Student's *t* test. (e) Expression analyses of *AaARF1*, *AaADS*, *AaCYP71AV1*, *AaALDH1*, and *AaDBR2* in WT, suppressed lines, and overexpression lines. Heatmaps show the significantly differential expression of genes (*P* < 0.05) in *A. annua*. Rows represent differentially expressed artemisinin biosynthesis genes, and columns represent group comparisons. Red indicates higher levels of gene expression and green indicates lower levels. The absolute signal intensity ranges from 0 to +1.0, with corresponding colour changes from green to red.

To further characterize the role of miR160 in *A. annua*, *MIR160* overexpression and suppression constructs were transferred into *Agrobacterium* EHA105 and genetically transformed into *A. annua*. In total, 13 independent *35S:miR160* lines and 15 independent *MIR160* knockdown lines (named STTM160) were obtained (Figure 4a,b). The transcript abundance of *MIR160* in selected transgenic *A. annua* plants was detected using qPCR. Reasonably, upon the comparison of the miRNA abundance with WT plants, the expression level of *MIR160* was found to be upregulated in the *35S:miR160* lines, while that of miR160 was reduced in the STTM160 lines (Figure 4c). The RNA‐Seq data showed that the expression levels of *AaADS*, *AaCYP71A1*, *AaALDH1*, and *AaDBR2* were significantly reduced in the *35S:miR160* lines. Conversely, the expression levels of these four enzymes were greatly increased greatly in the miR160 repression lines compared with the control (Figure 4e). Moreover, overexpressing miR160 dramatically decreased glandular trichome density, and the autofluorescence of many glandular trichomes exhibited a strong reduction on the *35S:miR160* plant leaves (shown by red arrows), indicating defective glandular trichomes (Figure 3b). The most severe reduction in glandular trichome density correlated with the highest expression level of *35S:miR160*‐7, while the glandular trichome density of STTM160 plants was increased significantly and showed strong green autofluorescence (shown by white arrows) compared with the controls. Consistent with the downregulation of genes involved in artemisinin biosynthesis, liquid chromatography–tandem mass spectrometry (LC–MS/MS) analysis showed that the artemisinin contents in *35S:miR160* lines decreased by 40%–70% compared with WT. In contrast, the artemisinin contents in STTM160 lines increased by 65%–81% compared with WT plants (Figure 4d). These results indicate that miR160 is a key regulator of glandular trichome formation and artemisinin biosynthesis.

### ARF1 mRNA cleavage is directed by miR160

**Figure 5:**
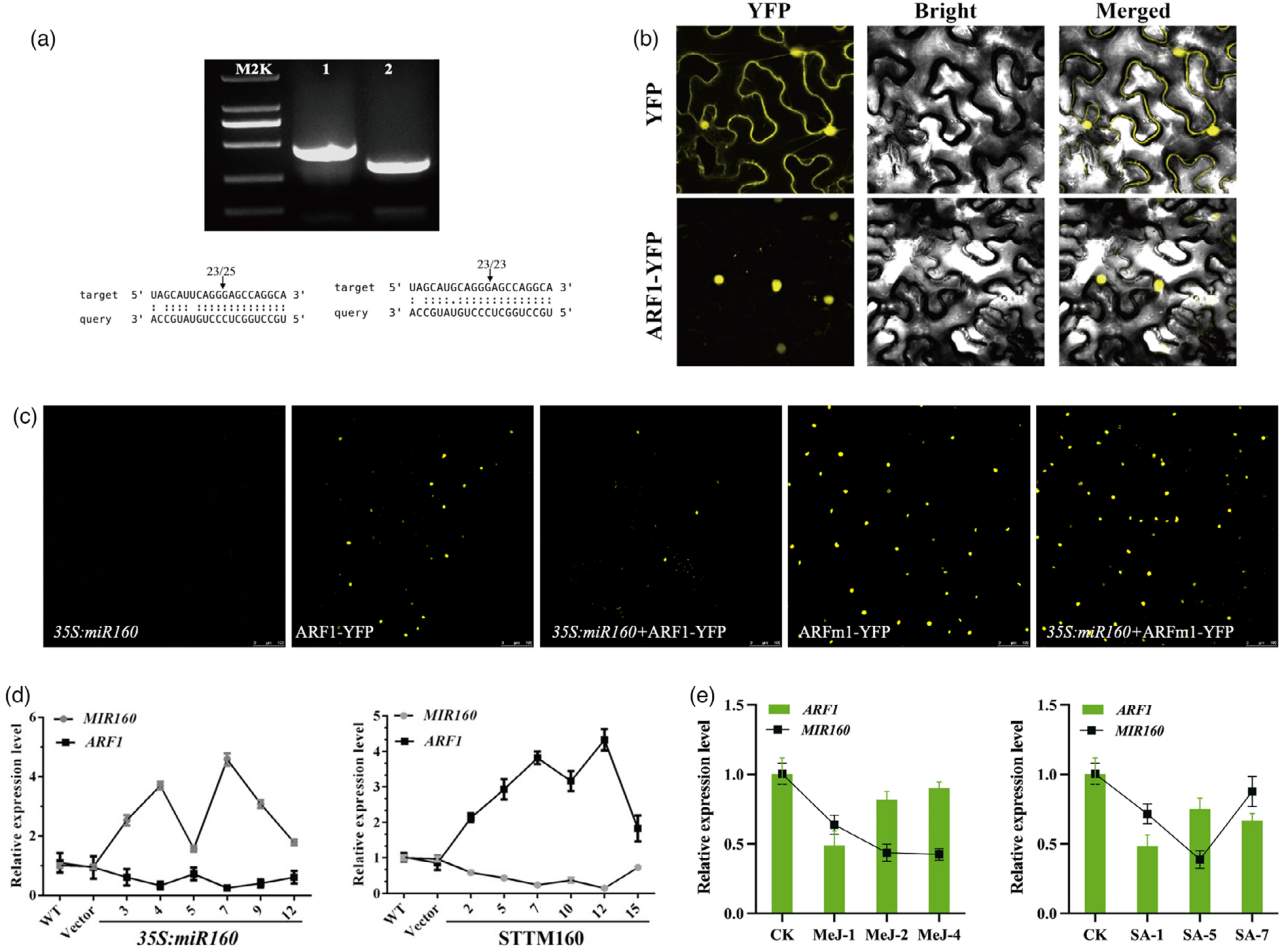
Experimental validation of *ARF1* as a target gene of miR160. (a) Nested PCR products of 5′ RACE. Vertical arrows indicate the 5′ termini of the miRNA‐guided cleavage products, as identified by 5′ RACE, with the frequency of clones shown. (b) Subcellular localization of ARF1. YFP was used as a negative control. Three independent transfection experiments were performed. (c) miR160 targeting of ARF1 was verified in *Nicotiana benthamiana* leaves. The indicated constructs were transformed or cotransformed into *N. benthamiana* leaves, and the expression of ARF1 was imaged. Experiments were performed three times. (d) The expression profiles of *MIR160* and the target gene *ARF1* by qPCR in *35 S:miR160* and STTM160 transgenic *A. annua* lines. All data represent the means ± SDs of three replicates from three cutting propagations. (e) The expression profiles of *miR160* and the target gene *ARF1* by deep sequencing in treated *A. annua*. The green bar represents the *ARF1*, and the black lines represent *miR160*. All data represent the means ± SDs of three replicates from three cutting propagations. The data are represented as the mean plus SD of *n* = 3 biological replicates. *P < 0.05, **P < 0.01, Student's *t* test.

To investigate the underlying mechanisms by which miR160 regulates artemisinin biosynthesis, we tried to identify its target genes. Seven target genes (AA320180, AA003210, AA004030, AA122040, AA203930, AA487720, and AA072530) were found to have been cleaved at their miR160‐specific cleavage sites according to the available *A. annua* degradome sequencing data, indicating that they could be the target genes of miR160 (Figure S3). All of the above seven target genes encode ARF family proteins with ARF motifs and are named ARF1 to ARF7. A phylogenetic analysis based on protein sequences showed that the predicted target genes are highly conserved and can be divided into two clades (Figure S4). Predicted miRNA targets were then validated using RNA ligase‐mediated 5′ RACE PCR. The cleavage products of *ARF1* and *ARF6* mRNA fragments generated by miR160 processing were successfully detected (Figure 5a). Sequence analysis of the amplified products from 25 independent cDNA clones suggested that the cleavage sites were located in the middle of the miR160 complementary region. The cleavage site of the miR160/*ARF1* pair was between the 10th and 11th nucleotides of miR160, whereas *ARF6* was mapped to the paired miR160 at the 9th or 10th nucleotide from the 5′‐end (Figure 5a). In conclusion, these results demonstrate that miR160 may regulate divergent aspects of artemisinin biosynthesis by targeting *ARF* genes.

To verify the preferential targeting of *ARF*s by miR160, we transiently performed a yellow fluorescent protein (YFP)‐based reporter assay in *Nicotiana benthamiana* as described in a previous report (Wang *et al*., 2014). As a negative control, we used a primer to introduce six mismatched nucleotides in the miR160/ARF mRNA complementary region of the *ARF* mRNA sequence without changing the encoded amino acid sequences, and the mutant was designated ARFm (Figure S5). Constructs harbouring ARF or ARFm fused with YFP under the control of the CaMV35S promoter were transiently transformed (either individually or with *35S:miR160*) into *N. benthamiana* leaf cells by infiltration. The expression of ARF‐YFP was evaluated by confocal microscopy. In contrast to YFP, which was distributed throughout the cell, a strong YFP signal was detected only in the nuclei of the transformed cells expressing ARF1 or ARFm1 (Figure 5c), suggesting that ARF1 has a putative role in the control of transcription (Figure 5b). As shown in Figure 5c, when ARF1 and miR160 were coexpressed in tobacco leaf epidermal cells, YFP signals were substantially reduced, showing that miR160 inhibits the expression of ARF1. In contrast, the YFP signals were not greatly affected in cells coexpressing ARF1m6‐YFP and miR160. Oppositely, miR160 did not silence the potential target gene ARF6 (Figure S6). Notably, the level of the *MIR160* transcript was highest in the transgenic line *35S:miR160*‐5, while the expression of *ARF1* was most reduced. The expression of *ARF1* was also increased significantly in the STTM lines (Figure 5d). The inverse expression pattern of *MIR160* and *ARF1* was also observed after phytohormone treatments (Figure 5e). In summary, these data suggest that miR160 may regulate artemisinin biosynthesis through its target gene *ARF1*.

### ARF1 positively regulates artemisinin biosynthesis by activating *AaDBR2* expression

**Figure 6:**
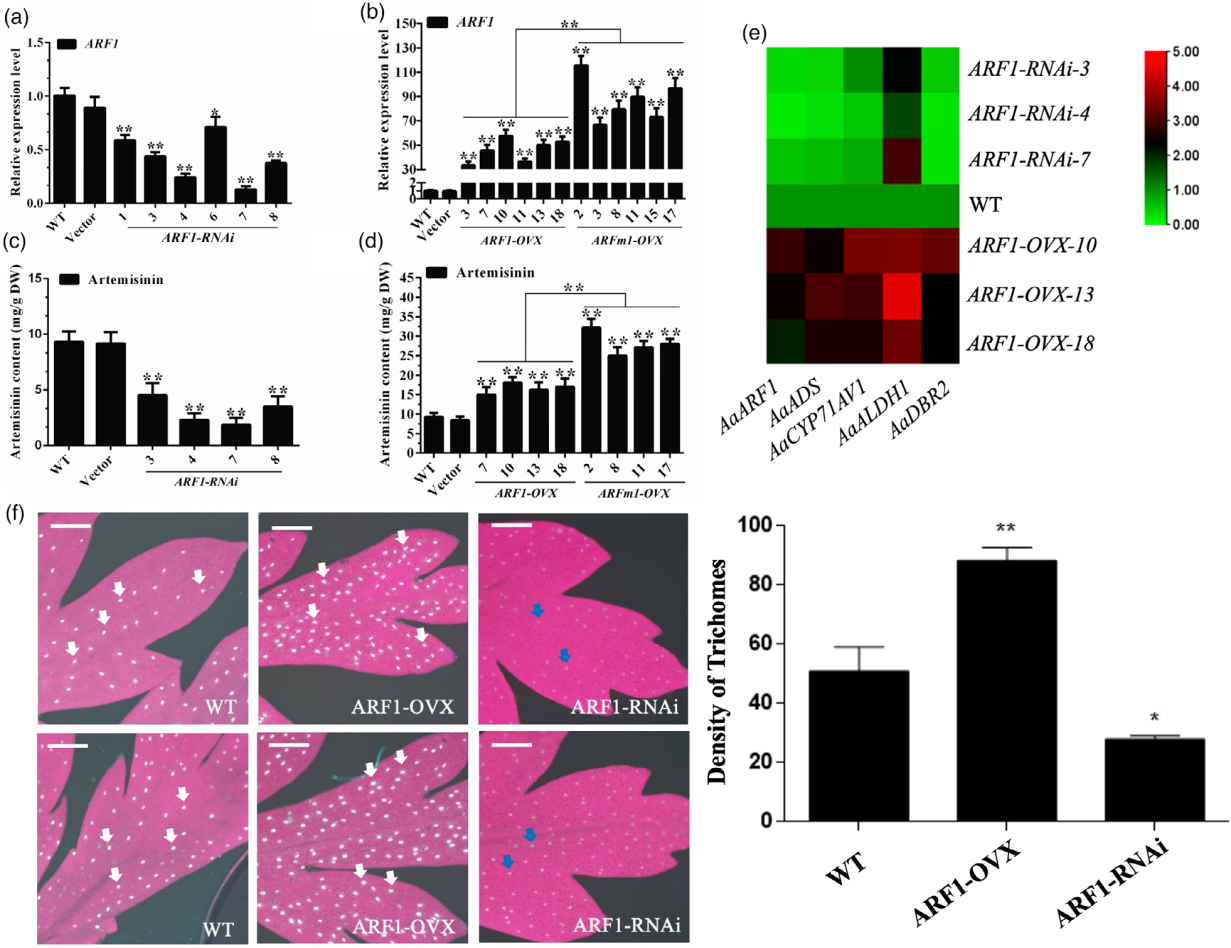
Analysis of *ARF1* transgenic plants. (a) Expression levels of *ARF1* in different *Artemisia annua* plants including *ARF1*‐*RNAi* lines, and plants transformed with the empty vector. *Actin* was used as the internal standard. WT plants served as controls. All data represent the means ± SDs of three replicates from three cutting propagations. *P < 0.05, **P < 0.01, Student's *t* test. (b) Expression levels of *ARF1* in different *A. annua* plants including *ARF1*‐*OVX* lines, *ARFm1*‐*OVX* lines, and plants transformed with the empty vector. *Actin* was used as the internal standard. WT plants served as controls. All data represent the means ± SDs of three replicates from three cutting propagations. *P < 0.05, **P < 0.01, Student's *t* test. (c) HPLC analysis of artemisinin in the leaves of different *A. annua* plants, including *ARF1*‐*RNAi* lines, plants transformed with the empty vector, and WT plants. All data represent the means ± SDs of three replicates from three cutting propagations. *P < 0.05, **P < 0.01, Student's *t* test. (d) HPLC analysis of artemisinin in the leaves of different *A. annua* plants, including *ARF1‐OVX* lines, *ARF1‐OVX* lines, plants transformed with the empty vector, and WT plants. All data represent the means ± SDs of three replicates from three cutting propagations. *P < 0.05, **P < 0.01, Student's *t* test. (e) Expression analyses of *AaARF1*, *AaADS*, *AaCYP71AV1*, *AaALDH1*, and *AaDBR2* in the WT, suppressed, and overexpression lines. Heatmaps show the significantly differential expression of genes (*P* < 0.05) in *A. annua*. Rows represent differentially expressed artemisinin biosynthesis genes, and columns represent group comparisons. Red indicates higher levels of gene expression and green indicates lower levels. The absolute signal intensity ranges from 0 to +5.0, with corresponding colour changes from green to red. (f) The adaxial surface was observed using fluorescence microscopy of leaves and the density of glandular trichomes in leaves from ARF1‐OVX and ARF1‐RNAi transgenic *A. annua* lines and WT plants. Bars represent 250 μm. *P < 0.05, **P < 0.01, Student's *t* test.

The results of 5′ RACE and transient expression experiments prompted us to investigate the effect of ARF1 on artemisinin biosynthesis. Given that miR160 overexpression results in reduced artemisinin production (Figure 4d), RNA interference (RNAi) of ARF1 was performed to assess whether ARF1 knockdown would produce the same phenotype. As shown in Figure 6a, the abundances of *ARF1* transcripts were confirmed to be greatly reduced by 41%–87% in ARF1‐RNAi plants, and four independent lines (ARF1‐RNAi‐3, ARF1‐RNAi‐4, ARF1‐RNAi‐7, and ARF1‐RNAi‐8) were chosen for further metabolism analysis. As expected, the contents of artemisinin in ARF1‐RNAi plants were reduced by 18%–44% (Figure 6c).

To further investigate whether miR160 regulates artemisinin by negatively regulating *ARF1* expression, *ARF1* or its mutant sequence *ARFm1* with six mismatches to miR160 were overexpressed. *ARF1* mRNA levels increased <57.6‐fold in ARF1‐OVX plants but were 66.7–115.5 times higher in ARFm1‐OVX plants than in empty vector controls (Figure 6b). In agreement with the *ARF1* transcript level, metabolism analysis showed that the ARFm1‐OVX lines accumulated higher artemisinin contents than the ARF1‐OVX lines. The artemisinin contents increased by 61%–94.6% and 168.8%–246.2% in the ARF1‐OVX and ARFm1‐OVX lines compared with WT lines, respectively (Figure 6d). Based on our RNA‐Seq data, the expression levels of artemisinin pathway genes (*AaADS*, *AaCYP71A1*, *AaALDH1*, and *AaDBR2*) were significantly enhanced after *ARF1* overexpression. Meanwhile, compared with the WT, the expression levels of these four enzymes were greatly reduced in ARF1‐RNAi lines (Figure 6e). Furthermore, overexpressing *ARF1* also increased glandular trichome density and the autofluorescence of glandular trichomes on ARF1‐OVX plant leaves (shown by white arrows). However, compared with the WT lines, the glandular trichome density of ARF1‐RNAi plants was decreased significantly and showed weak green autofluorescence (shown by blue arrows) (Figure 6f). These results confirm that *ARF1* is the target of miR160 and is intimately involved in the regulation of glandular trichome formation and artemisinin biosynthesis.

**Figure 7:**
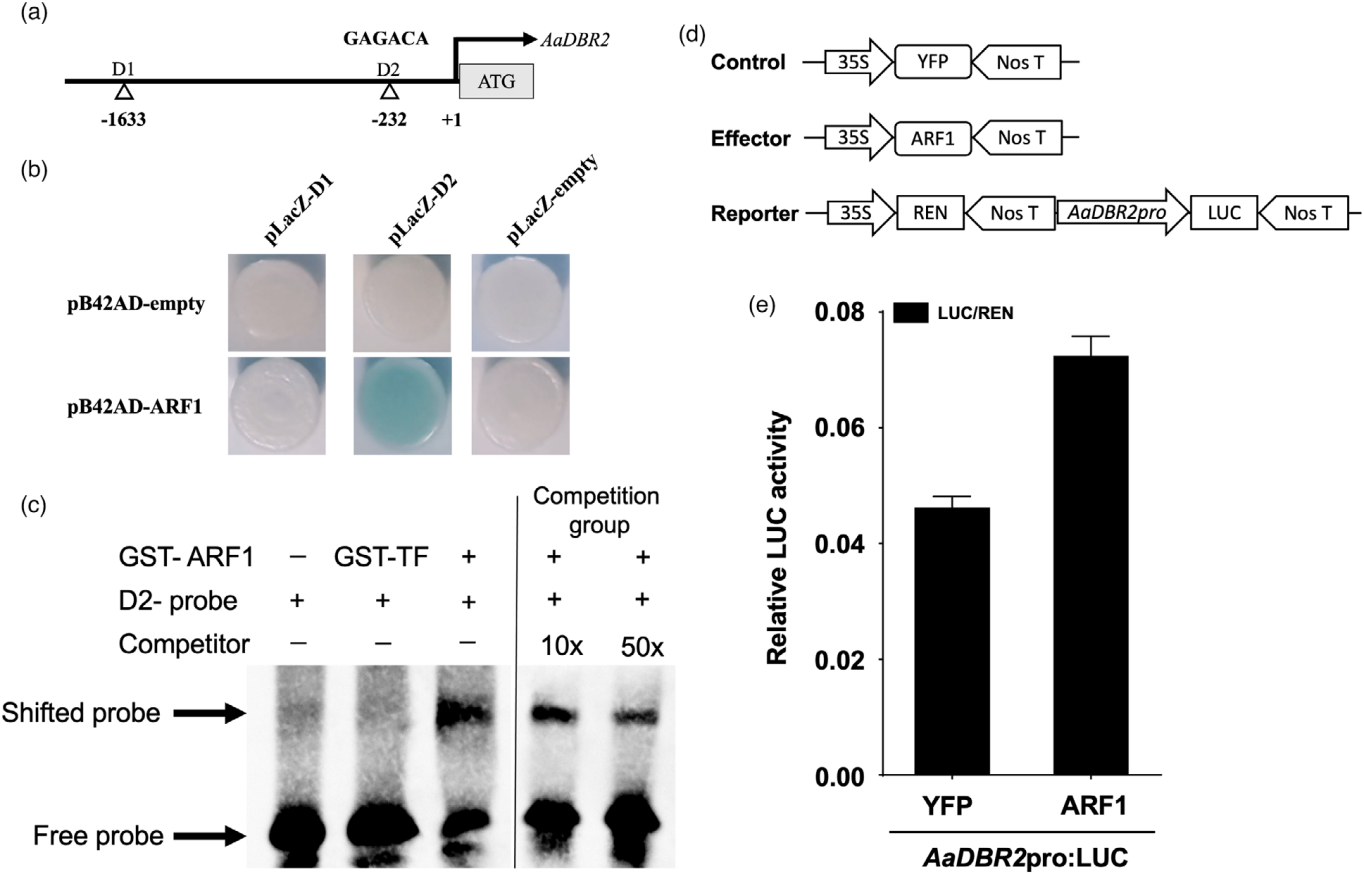
ARF1 directly activates the expression of AaDBR2. (a) Schematic diagrams of the *AaDBR2* promoters. The black triangles represent the positions of the ‘GAGACA’ cis‐elements in the *AaDBR2* promoter. (b) Yeast one‐hybrid assay of protein‐DNA interactions. Empty vectors were used as a negative control. Representative results are shown. (c) EMSA showing ARF1 binding to the second GAGACA box of the *AaDBR2* promoter. An unlabeled D2 probe was used as a cold competitor, and GST‐TF protein was used as a negative control. The fold excesses of cold competitors relative to that of the labelled probe are indicated as 10× and 50×. The EMSAs were also repeated three times, and representative results are shown. (d) Schematic representation of the control, effectors, and reporters used in the dual‐LUC assays. (e) LUC reporter constructs harbouring *AaDBR2* promoters were used as reporters (*AaDBR2*pro:LUC). Effector constructs harbouring full‐length ARF1 were driven by the CaMV35S promoter. The LUC activities were normalized to Renilla (REN) luciferase activities. The YFP driven by the 35S promoter was used as a negative control.

To investigate the underlying mechanisms by which ARF1 regulates artemisinin biosynthesis, we performed a yeast one‐hybrid assay (Y1H), an electrophoretic mobility shift assay (EMSA), and transient dual‐luciferase (dual‐LUC) analysis. ARF TFs are reported to commonly bind to the GAGACA box (AuxRE) of the promoter region and achieve subsequent promotion or repression of expression (Ulmasov *et al*., 1999). To identify the potential binding sites of ARF1, the Plant CARE cis‐regulatory element database (http://bioinformatics.psb.ugent.be/webtools/plantcare/html/) was used to analyse the promoters of artemisinin pathway genes (*AaADS*, *AaCYP71AV1*, *AaALDH1*, and *AaDBR2*), which showed that only *AaADS* and *AaDBR2* promoters contain a putative AuxRE. Y1H assays showed that the binding of the *p*B42AD‐ARF1 fusion protein, but not *p*B42AD alone, to three tandem repeats of the D2 motif strongly activated the expression of the *LacZ* reporter gene (Figure 7a,b), indicating that ARF1 binds to the D2 motif of the *AaDBR2* promoter *in vivo*. However, interactions between ARF and the promoters of the *AaADS* gene were not detected in Y1H assays, implying that ARF might fulfil its positive regulatory function in part by interacting with *AaDBR2*.

Furthermore, EMSAs were performed (Figure 7c), and a single shifted band was observed only in the presence of both GST‐ARF1 and the labelled DNA probe containing the D2 motif. The intensity of the shifted band decreased with increasing concentrations of a cold competitor, and no band was observed when the control protein GST‐TF was added in place of GST‐ARF1. The results of EMSAs indicated that ARF1 can bind to the D2 motif of the *AaDBR2* promoter *in vitro*. In dual‐LUC assays, when ARF1‐YFP was transiently expressed in *N. benthamiana* leaf cells harbouring the DBR2pro:LUC plasmid, the promoter activities of *AaDBR2* significantly increased compared with the YFP control (Figure 7d,e). These results demonstrate that ARF1 positively regulates *AaDBR2* expression by directly binding to its promoters. In conclusion, this study illustrates that miR160 negatively regulates artemisinin synthesis by degrading the positive TF ARF1 of the artemisinin pathway gene *AaDBR2*.

## Discussion

Plant miRNAs play an important regulatory role in secondary metabolism by targeting mRNA degradation (Samad *et al*., 2017). Although miRNA‐TF modules have been shown to be critical for regulating secondary metabolic synthesis, the regulatory mechanism of artemisinin synthesis has not been elucidated (Samad *et al*., 2017). Phytohormone treatments, including MeJA, ABA, and SA, have been reported to increase the artemisinin content in *A. annua* by both elevating the biosynthetic levels and the glandular trichome density (Xiao *et al*., 2016). Thus, we systematically mapped phytohormone‐responsive miRNA profiles in *A. annua* for the first time, intending to broaden our understanding of the biological functions of miRNAs in the biosynthesis of artemisinin. Many novel phytohormone‐specific miRNAs, which have not been reported in previous works (Khan *et al*., 2020; Pani *et al*., 2011; Perez‐Quintero *et al*., 2012) were successfully discovered. In total, we identified 47 conserved miRNA families in the 10 libraries, of which miR156 was the largest (Figure 1c). This finding is consistent with recent reports of salt stress‐regulated miRNAs in *Medicago sativa* (Long *et al*., 2015) and drought‐responsive miRNAs in *Camellia sinensis* (Guo *et al*., 2017). The lower abundance and fewer identified targets of novel miRNAs compared to conserved miRNAs suggest that the majority of them may not be functional (Xia *et al*., 2022), whereas the abundantly expressed conserved miRNAs may be the dominant small RNA regulators of artemisinin biosynthesis.

It is well known that miRNAs work by suppressing the expression of target genes. Genome‐wide analysis of the degradome was performed, and numerous target transcripts of known and novel miRNAs were identified. To assess the miRNA‐mediated regulation of artemisinin biosynthesis, we searched specifically for miRNA targets of identified miRNAs among annotated genes of the artemisinin biosynthesis pathway. However, no miRNA targets were found among these pathway genes, which implied that the regulation of the artemisinin pathway by miRNA is not direct but may be achieved through the miRNA‐TF module. We then predicted that some miRNAs might regulate artemisinin biosynthesis through phytohormone signal transduction during hormone stress. However, their exact functions remain to be verified in future investigations.

miR160 was the most abundant in our sequencing samples and was differentially expressed in response to MeJA and SA treatments (Figure 1f). Overexpression of miR160 resulted in decreased glandular trichome density and artemisinin content in *A. annua*, which reveals that miR160 mediates the regulation of glandular trichome development and artemisinin biosynthesis. Unexpectedly, miR160 also exhibits functions involved in trichome development in *A. thaliana*, although the regulatory patterns of glandular trichome and nonglandular trichome development are generally believed to be different.

The members of the miR160 family are reported to be able to regulate the auxin signal transduction pathway by targeting genes of the ARF family and play central roles in plant growth and development (Hao *et al*., 2022). In *A. thaliana* and tomato (*Solanum lycopersicum*), multiple ARF TFs are downregulated by miR160 through a translational mechanism (Hendelman *et al*., 2012; Li *et al*., 2016; Remington *et al*., 2004). In *A. thaliana*, ARF10/16/17, which repress auxin activity, is important for the development of reproductive and vegetative tissues under the control of miR160 (Dai *et al*., 2021). Additionally, miR160 targets ARF10/16/17 to regulate the auxin/cytokinin balance during nodule formation in soybean (Nizampatnam *et al*., 2015). Overexpression of *SlARF10* has been shown to alter leaf size resulting in leaflets with extremely narrow blades (Yuan *et al*., 2018). Auxin‐responsive ARF4 is highly expressed in type II, V, and VI trichomes and positively regulates trichome formation in tomato leaves, although it has not been shown to be regulated by miR160 (Yuan *et al*., 2021). Our RACE results demonstrated direct cleavage of target mRNAs by miR160 (Figure 5a), but we cannot exclude the possibility that miR160 may also regulate its target translational repression at the post‐transcriptional level. In this study, we found that *ARF1* was a positive regulator of artemisinin biosynthesis and glandular trichome formation in *A. annua*. We found that ARF1 could affect artemisinin biosynthesis by directly regulating the expression of *DBR2* in the artemisinin pathway. However, the mechanism of its regulation of glandular trichome development remains to be further explored.

**Figure 8:**
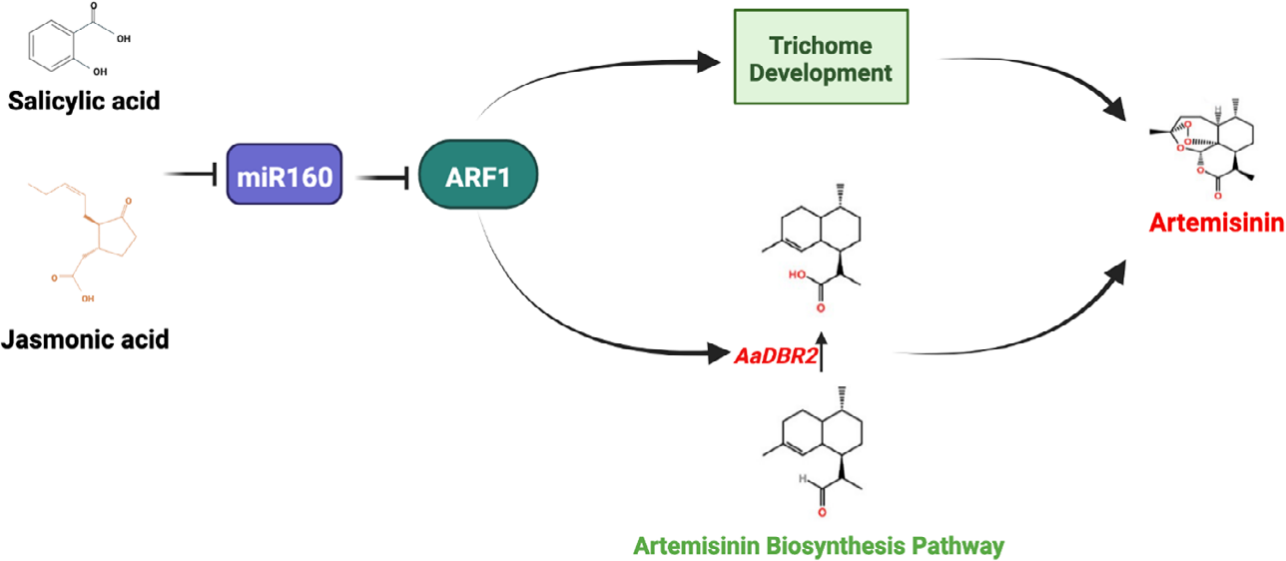
Proposed model for the regulatory roles of the miR160‐ARF1 module involved in artemisinin biosynthesis in *Artemisia annua*.

As in *A. thaliana* and tomato, ARFs always function in the transcriptional regulation of secondary metabolism with miR160 building blocks (Shen *et al*., 2017; Wang *et al*., 2020). However, there is no evidence of a direct link between the module and the biosynthesis of artemisinin. In this study, we provide solid evidence that miR160‐ARF1 in *A. annua* regulates glandular trichome development and artemisinin biosynthesis in a module fashion (Figure 8). Our results provide novel insights into the miRNA‐mediated regulation of gene expression and artemisinin biosynthesis in *A. annua*. However, the mechanism by which the miR160‐ARF1 module responds to phytohormones and regulates glandular trichome development still needs to be further explored to fill the gap in knowledge of the phytohormone regulation network involved in artemisinin synthesis (Lantzouni *et al*., 2020; Lin *et al*., 2015). Furthermore, in addition to the artemisinin pathway gene *DBR2*, whether there is a regulatory relationship between the miR160‐ARF1 module and other well‐defined artemisinin synthesis‐related TFs (such as *AaHD1*, *AaHD8*, and *AaMIXTA1*) also deserves further exploration. In conclusion, this study clarified the biological functions of the miR160‐ARF1 module in regulating artemisinin synthesis and glandular trichome development, supplemented the regulatory network of phytohormone‐induced artemisinin synthesis, and provided a new idea for the breeding of high‐quality *A. annua*.

## Methods

### Plant materials

The high‐artemisinin cultivar of *A. annua* L., named ‘Huhao 1’, which originated from Chongqing was used for all *A. annua*‐related assays (Lv *et al*., 2019). The seeds were first surface‐sterilized with 70% ethanol for 1 min, followed by 10% sodium hypochlorite solution for 10 min, and then rinsed four times with sterile water. After that, the seeds were sown on Murashige and Skoog (MS) medium (Sigma–Aldrich) with 88 mm sucrose and 0.7% agar (pH 5.8) and incubated with a photoperiod of 16/8 h light/dark with 7500 lux at 26 °C.

The *Arabidopsis* (*A. thaliana*) WT and transgenic plants used in this work were Col‐0 ecotypes grown in pots in a growth chamber under a 24 °C and 16‐h light/8‐h dark photoperiod. For *in vitro* culture, seeds were surface‐sterilized in 10% sodium hypochlorite solution and 0.01% Triton X‐100 for 5 min and washed three times in sterile distilled water. After conducted for 3 d at 4 °C, the seeds were sown on plates containing MS solid medium composed of MS basal salts and 20% sucrose solidified with 0.6% agar at pH 5.7. The plates were sealed and incubated in a controlled‐environment growth chamber.

### Hormone treatments

For hormone treatments, 30‐day‐old *A. annua* seedlings were sprayed with MeJA (100 μm), ABA (100 μm), or SA (100 mm), whereas water with 1‰ concentration of DMSO was used as a mock treatment (CK). Seedling samples were collected at 1, 2, and 4 h after spraying with MeJA, 1, 5, and 7 h after spraying with SA, and 2, 6, and 12 h after spraying with ABA. These treated samples were denoted MeJ_1H, MeJ_2H, MeJ_4H, SA_1H, SA_5H, SA_7H, ABA_2H, ABA_6H, and ABA_12H. To reduce the differences between individuals, at least 50 seedlings were collected as samples at a single time point. For each group, three individual seedlings were used as biological repeats. Pooled seedling samples were collected and frozen in liquid nitrogen immediately and stored at −80 °C for further analysis.

### Transcriptome sequencing and *de novo* assembly analysis

Total RNA was extracted using the Total RNA Purification Kit, TRK1001 (LC Sciences, Houston, TX, USA). All RNA samples were treated with DNase I (TaKaRa, Dalian, China) to avoid genomic DNA contamination. RNA quality and purity were checked using denaturing 1.0% (p/v) agarose gel electrophoresis and a NanoDrop 2000 spectrophotometer (Thermo Fisher Scientific, Waltham, MA, USA) at 260/280 nm (ratio >2.0). The total RNA quantity and purity were analysed using an Agilent 2100 Bioanalyzer and an RNA 1000 Nano LabChip Kit (Agilent, CA, USA) with RIN >7.0. mRNAs with poly (A) tails were purified from the total RNA, using oligo (dT) magnetic beads, and then fragmented with an RNA fragmentation kit with two rounds of purification. Then, the cleaved RNA fragments were reverse‐transcribed to create the final cDNA library following the protocol for the TruSeq Stranded mRNA Library Prep Kit (Illumina, San Diego, CA, USA), and the average insert size for the paired‐end libraries was 300 bp (±50 bp). Paired‐end sequencing was performed on an Illumina HiSeq2000 (LC Sciences) following the vendor's recommended protocol. *De novo* assembly of the transcriptome was performed with Trinity. Trinity groups transcripts into clusters based on shared sequence content. Such a transcript cluster is very loosely referred to as a ‘gene’. The longest transcript in the cluster was chosen as the ‘gene’ sequence (aka Unigene).

### Small RNA sequencing and miRNA identification

Total RNA from the aerial tissue of *A. annua* of the control and treated samples was extracted using the EASYspin Plus Plant RNA Kit (Aidlab Bio, Beijing, China) according to the manufacturer's protocol. Ten small RNA libraries (CK, MeJ_1H, MeJ_2H, MeJ_4H, SA_1H, SA_5H, SA_7H, ABA_2H, ABA_6H, and ABA_12H) were constructed by Solexa/Illumina sequencing (LC Bio, Hangzhou, China). The raw RNA reads generated by next‐generation sequencing (NGS) from the 10 sRNA libraries were processed to remove 5′ and 3′ adapters and contaminated and low‐quality sequences, as well as those smaller than 18 nt, through Illumina's Genome Analyzer Pipeline V1.5. The filtered reads were subjected to a further filtration step to remove the common RNA families (rRNA, tRNA, and snRNA) with a proprietary pipeline script ACGT101‐miR v4.2 (LC Sciences). Then, the remaining clean and unique reads were aligned against the latest miRBase database, version 22.0 (http://www.mirbase.org/), using the BLAST algorithm to identify known miRNAs. The stem‐loop hairpin structures were aligned to sequencing reads and mature miRNAs from miRBase using Bowtie. Ten groups of miRNAs were revealed through bioinformatics analysis of sRNA sequencing based on the classification method. The read distribution was checked to meet the principles for miRNA prediction and authentic miRNAs were regarded as described previously (Taylor *et al*., 2014). The miRNA sequencing raw data are available at NCBI SRA (BioProject ID: PRJNA756118).

### Degradome sequencing, target identification, and analysis

Equal amounts of RNA samples treated with MeJA, ABA, or SA at different time points were mixed to generate 10 degradome libraries. The extracted sequencing reads with lengths of 20 and 21 nt were then used to identify potentially cleaved targets by the Cleveland pipeline. Then, the degradome reads were mapped to the *A. annua* genome data. The targets selected were categorized as 0, 1, 2, 3, and 4 as in a previous study (Addo‐Quaye *et al*., 2008). Based on the signatures (and abundances) along the hormone‐treated *A. annua* transcriptome data, t‐plots were built for high‐efficiency analysis of the potential miRNA targets. Finally, all of the identified potential target genes were subjected to an NCBI search using the BLASTX algorithm and GO analysis. The degradome sequencing raw data are available at NCBI SRA (BioProject ID: PRJNA756886).

### Differentially expressed target gene analysis

To discover the expression profiles of the target genes, 10 independent libraries were constructed from each of the RNA samples from 10 different hormone treatment durations. For each library, all of the sequences were processed to filter out the adapter and low‐quality sequences. Then, all of the clean tags were mapped to the assembled unigenes of *A. annua* for annotation. The reads per kb per million reads method was used to calculate the gene expression level. Then, a rigorous algorithm method was performed to identify the differentially expressed genes between the two samples. The false discovery rate method was used to determine the *P* value threshold in multiple tests and analyses. The significantly differentially expressed genes among all of the different samples were judged by the following thresholds: *P* value <0.005, false discovery rate ≤0.001, and the absolute value of log2 ratio ≥1. The heatmap of the differentially expressed miRNAs was constructed using the ggplot2 package in R (version 3.1.3).

### RNA extraction and quantitative PCR analysis

Total RNA was extracted from the young leaves of *A. annua* plants using the TransZol Up Plus RNA Kit (Transgene, Beijing, China) and total small RNAs were extracted from *A. annua* using the miRNA Isolation Kit (Invitrogen, Carlsbad, CA, USA). One microgram of RNA was used to prepare first‐strand cDNA using TransScript First‐Strand cDNA Synthesis SuperMix (Transgene). First‐strand cDNA was also synthesized from the total small RNAs using a miRNA First Strand cDNA Synthesis Kit (Sangon, Shanghai, China). qRT‐PCR was performed on a Dice Real‐Time PCR machine (TaKaRa, Tokyo, Japan) using the TransStart Top Green qPCR SuperMix Kit (Transgene) according to the manufacturer's instructions. Stem‐loop‐specific reverse transcription for miRNAs was performed as described previously (Chen *et al*., 2005). The relative expression levels of genes were normalized to the expression of *A. annua* Actin. All gene expression data are from three biological replicates with three technical replicates for each biological sample. All primers used for stem‐loop qRT‐PCR are listed in Table S4.

### Prediction of miRNA targets and 5′ RACE mapping of miRNA target cleavage sites

A modified procedure for RNA ligase‐mediated rapid amplification of 5′ cDNA ends (RLM‐5′ RACE) was conducted with the FirstChoice RLM‐RACE kit (Invitrogen) to map the cleavage sites of target transcripts. Briefly, 250 μg of a mixture of total RNA from the aerial parts of *A. annua* were subjected to an Oligotex mRNA Mini Kit (Qiagen, Hilden, Germany) for poly(A) mRNA isolation. Poly(A) mRNA was directly ligated to the 5′ RACE RNA Oligo adapter (45 nucleotides) from the FirstChoice RLM‐RACE Kit without alkaline phosphatase and tobacco acid pyrophosphatase treatment. The oligo (dT) (15‐mer) primer was used to synthesize cDNA with reverse transcriptase. The resulting cDNA samples were amplified by nested PCR according to the manufacturer's protocols. ARF outer and inner primers were designed for the lateral outer and inner PCRs (Table S5). Inner PCR products were cloned into the *Trans1*‐*T1* vector (Transgene) and sequenced. For each target, 25 single clones were sequenced.

### Plasmid construction

For the miR160 overexpression construct, the sequence (153 bp) containing the *MIR160* foldback was amplified from *A. annua* genomic DNA and inserted into the plant expression vector PHB‐flag under the control of the 35S promoter of cauliflower mosaic virus (CaMV) using *Spe*I and *Bam*HI. The resulting construct was verified by DNA sequencing and named *35S:miR160*. The STTM160 sequence was constructed to silence the activity of miR160, which contained two copies of imperfect miR160 binding sites with a 48 nt linker, and each copy had a cleavage‐preventive bulge containing three additional nucleotides (CTA) that was made using the method according to the reference (Yan *et al*., 2012). The STTM160 module was inserted between the 35S promoter and the 35 S terminator in the PHB‐flag vector, and the resulting construct was named STTM160.

To verify the direct target genes of miR160 and analyse the subcellular localization of the target genes, the open reading frames (ORF) of *ARF1* and *ARF6* were amplified and inserted into the expression vector PHB‐YFP using *Hin*dIII and *Bam*HI under the CaMV35S promoter to generate PHB‐ARF1‐YFP or PHB‐ARF6‐YFP fusion protein.

For site‐directed mutagenesis, six‐point mutations of ARF1 and ARF6 in the nucleotide sequences of the miR160 complementary sites were designed according to the procedure of Chen using the Hieff Mut™ Multi Site‐Directed Mutagenesis Kit with *ARF1* or *ARF6* mutagenesis forward and reverse primers (Table S6) (Yeasen, Shanghai, China) (Chen, 2004). The resulting clones were designated PHB‐ARFm1‐YFP and PHB‐ARFm6‐YFP, respectively. Correct mutagenesis was verified by sequencing. For ARF1‐overexpression vector construction, the coding region of *ARF1* was amplified and inserted into the vector PHB‐flag using the *Spe*I and *Bam*HI restriction sites to generate the ARF1‐PHB construct. To construct the *ARF1* RNAi vector, a less conserved region at the C‐terminus of ARF1 (510 bp) was amplified by PCR from ARF1 cDNA. The fragment was placed in forward and reverse orientation on the two ends of the pyruvate orthophosphate dikinase intron to generate the ARF1‐RNAi construct. All primers used for plasmid construction are listed in Table S3.

### *Agrobacterium tumefaciens* infiltration in *Nicotiana benthamiana*

The plasmids PHB‐ARF1‐YFP, PHB‐ARF6‐YFP, PHB‐ARFm1‐YFP, and PHB‐ARFm6‐YFP were transformed into *A. tumefaciens* strain GV3101 and transiently infected into epidermal cells of *N. benthamiana*. Infiltration and detection were performed according to the protocols described previously with minor modifications (Ma *et al*., 2018). The transformed *A. tumefaciens* cells were resuspended in MS liquid medium buffer with 10 mm methylester sulfonate and 150 μm acetosyringone at OD_600_ = 0.6 and incubated at room temperature for at least 3 h before being infiltrated into the abaxial air spaces of 5‐week‐old *N. benthamiana* plants. For the coinfiltration experiments, equal volumes of an *Agrobacterium* culture containing *35S:miR160* (OD_600_ = 1.75) and PHB‐ARF1‐YFP, PHB‐ARF6‐YFP, PHB‐ARFm1‐YFP, or PHB‐ARFm6‐YFP (OD_600_ = 0.25) were mixed before infiltration into *N. benthamiana* leaves. After incubation at 23 °C for 60 to 72 h, YFP signals were observed with a Leica TCS SP5 confocal laser scanning microscope (Leica Microsystems, Wetzlar, Germany). The PHB‐YFP construct was used as the negative control. Three biological repeats were performed to verify these results.

### Plant transformation and phenotype analysis

The resulting plasmids *35S:miR160* and STTM160 were introduced into the *Agrobacterium strain* GV3101, and *A. thaliana* Col‐0 WT plants were transformed with these constructs using the *Agrobacterium*‐mediated floral dip method. Progeny from self‐fertilized primary transformants was grown in soil for observation of the glandular trichome phenotype.

The overexpression constructs *35S:miR160*, ARF1‐PHB, and STTM160; the construct version resistant to miR160 cleavage, ARFm1‐PHB; and the RNAi construct RNAi‐ARF1 were transferred into *A. tumefaciens* strain EHA105 as described above. The transformation of *A. annua* was carried out according to a previous description (Zhang *et al*., 2009). The phenotypes of *A. annua* plants transformed with the empty vector (control plants), *35S:miR160* overexpression and STTM160 silencing were observed at the indicated times under normal conditions. Fresh leaves were placed under a 488 nm excitation wavelength, and the glandular trichome fluorescent signal was imaged on an Olympus BX43 microscope. The total number of glandular trichomes was counted in a 1‐mm^2^ leaf area to measure the glandular trichome density using the ImageJ software.

### Measurement of artemisinin content using HPLC–MS/MS

Leaves were collected from 3‐month‐old *35S:miR160*, STTM160, ARF1‐PHB, ARFm1‐PHB, and RNAi‐ARF1 transgenic *A. annua* plants; *A. annua* plants were transformed with the empty vector; and WT plants grown in the greenhouse were dried at 50 °C overnight and ground to powder. Dried leaf powder (0.1 g) was extracted twice with 2 mL of methanol under ultrasound for 30 min. After centrifugation at 16 000 × *g* for 5 min, the supernatant was filtered through a 0.22‐μm microfiltration membrane. The concentrations of artemisinin in the final samples were measured by HPLC/MS–MS (Zhou *et al*., 2020).

### Yeast one‐hybrid assay

The Matchmaker Gold Yeast One‐Hybrid System (Clontech, Suzhou, China) was constructed to investigate the DNA binding properties of the ARF1 protein. The auxin‐responsive TGTCTC elements of the *DBR2* promoter were cloned into the *p*LacZ vector, and the ORFs of ARF1 were amplified and inserted separately into the yeast expression vector *p*B42AD. The empty *p*B42AD vector was used as a negative control. Different combinations were cotransformed into the yeast strain EGY48a. The cotransformed yeast cells were cultivated on the SD‐Trp‐Ura medium, and the SD‐Trp‐Ura medium with X‐gal was used as a selection medium. All primers are listed in Table S6.

### Electrophoretic mobility shift assay

The ARF1 ORF was cloned into the pGEX‐4T‐1 vector to produce GST‐tagged fusion proteins. The pGEX‐4T‐1‐ARF1 construct and the negative control empty pGEX‐4T‐1 vector were transformed into the *Escherichia coli* strain *Transetta* DE3 (TransGen, Beijing, China). Then, 0.75 mm isopropyl β‐d‐1‐thiogalactopyranoside was added to induce the expression of the fusion protein for 16 h at 16 °C after the *Transetta* DE3 strains were cultured to OD_600_ = 0.6. GST‐tag Purification Resin (Beyotime Biotech, Shanghai, China) was used to purify the fusion proteins from the pGEX‐4T‐1‐ARF1 and empty pGEX‐4T‐1 vectors.

The 50 bp biotin‐labelled oligonucleotides for the D2 elements were synthesized (Genewiz, Suzhou, China) and equimolar pairs were annealed. EMSAs were performed using the Light‐Shift Chemiluminescent EMSA Kit (Thermo) according to the manufacturer's instructions. Two micrograms of recombinant protein and 100 fmol biotin‐labelled DNA with binding reaction buffer were incubated for 20 min at 25 °C. An ultraviolet (UV) cross‐linker was used to cross‐link the DNA after blotting it on a positively charged nylon membrane. The biotin‐labelled DNA was detected by chemiluminescence and exposed to X‐ray film. The probes and primers used in the EMSA are listed in Table S6.

### Dual‐LUC assay

For the dual‐LUC assay, PHB‐ARF1 was transformed into *A. tumefaciens* strain GV3101 as an effector. The promoter of *DBR2* was fused to the firefly luciferase gene on the plasmid *p*GreenII 0800‐LUC into GV3101 to act as the reporter. Then, the incubated *Agrobacterium* cells were harvested by centrifugation and resuspended in MS medium (containing 10 mm MES and 150 mm acetosyringone) to an OD_600_ of 0.6. The reporter construct AaDBR2pro:LUC was mixed with the effector strain GV3101 harbouring 35Spro:ARF1 in a 1:1 ratio and injected into tobacco leaves after 3 h of incubation at room temperature. After incubation for 48 h under low‐light conditions, the leaf samples were collected for the dual‐LUC assay. The dual‐LUC assays were performed using the Promega Dual‐Luciferase Reporter Assay system according to the manufacturer's instructions. The probes and primers used in the dual‐LUC assay are listed in Table S6.

## Conflict of interest

The authors declare that the research was conducted in the absence of any commercial or financial relationships that could be construed as potential conflict of interest.

## Author contributions

Z.G., Z.L., K.H., H.Z., R.C., and L.Z. conceived and designed the entire research plans; Z.G., K.H., L.Y., J. R, R.C., and Z.L. performed most of the experiments; Z.L. and Q.B. provided technical assistance to Z.G., Z.G., K.H., Z.L., H.Z., R.C., and L.Z. wrote the manuscript.

## Supporting information


**Figure S1** GO analysis of putative miRNA target genes. GO annotation categorized all of the predicted miRNA target genes and differentially expressed miRNA target genes into biological processes, cellular components, and molecular functions.
**Figure S2** KEGG analysis of putative miRNA target genes. Functional annotation of KEGG pathways in *Artemisia annua* by the KEGG database.
**Figure S3** Target plots of the targets cleaved by miR160. The T‐plots show the distribution of the degradome tags along the full‐length target mRNA sequence. The red lines represent the predicted cleavage sites of the corresponding miRNAs. (A–G) Cleavage features of *ARF1*–*ARF7* mRNA by miR160 from the degradome library, respectively.
**Figure S4** Neighbour‐joining phylogenetic tree of ARF from *Artemisia annua*. Sequences were aligned using Clustal W, and the phylogenetic tree was constructed with MEGA. Bootstrap values were obtained for 1000 replications.
**Figure S5** The mutant nucleotides in *ARFm1* and *ARFm6* are shown in red. Although *ARFm6* causes a Met‐to‐Ile amino acid substitution (underlined codon), *ARFm6* does not change the amino acid sequence.
**Figure S6** miR160‐targeted ARF6 was verified in *Nicotiana benthamiana* leaves. 1, miR160‐PHB; 2, ARF6‐YFP‐PHB; 3, miR160‐PHB + ARF6‐YFP‐PHB; 4, ARFm6‐YFP‐PHB; 5, miR160‐PHB + ARFm6‐YFP‐PHB.

**Table S1** Small RNA deep sequencing profiles for *Artemisia annua* under MeJA, SA, and ABA treatments.

**Table S2** Novel miRNAs in *Artemisia annua*.

**Table S3** miRNA targets.

**Table S4** Stem‐loop primers of miRNA for reverse transcription, primers of miRNA for qRT‐PCR.
**Table S5** Primers for RLM‐5′ RACE.
**Table S6** Primers used for plasmid construction in this study.
