# Supplementary material for: Profiling of phytohormone‐specific microRNAs and characterization of the miR160‐ARF1 module involved in glandular trichome development and artemisinin biosynthesis in Artemisia annua
Source: Plant Biotechnol J. 2022 Dec 20;21(3):591–605. doi: 10.1111/pbi.13974 (PMC9946145; doi:10.1111/pbi.13974)
Supplement: Supplementary file 1 — Figure S1 GO analysis of putative miRNA target genes. GO annotation categorized all of the predicted miRNA target genes and differentially expressed miRNA target genes into biological processes, cellular components, and molecular functions. Figure S2 KEGG analysis of putative miRNA target genes. Functional annotation of KEGG pathways in Artemisia annua by the KEGG database. Figure S3 Target plots of the targets cleaved by miR160. The T‐plots show the distribution of the degradome tags along the full‐length target mRNA sequence. The red lines represent the predicted cleavage sites of the corresponding miRNAs. (A–G) Cleavage features of ARF1–ARF7 mRNA by miR160 from the degradome library, respectively. Figure S4 Neighbour‐joining phylogenetic tree of ARF from Artemisia annua. Sequences were aligned using Clustal W, and the phylogenetic tree was constructed with MEGA. Bootstrap values were obtained for 1000 replications. Figure S5 The mutant nucleotides in ARFm1 and ARFm6 are shown in red. Although ARFm6 causes a Met‐to‐Ile amino acid substitution (underlined codon), ARFm6 does not change the amino acid sequence. Figure S6 miR160‐targeted ARF6 was verified in Nicotiana benthamiana leaves. 1, miR160‐PHB; 2, ARF6‐YFP‐PHB; 3, miR160‐PHB + ARF6‐YFP‐PHB; 4, ARFm6‐YFP‐PHB; 5, miR160‐PHB + ARFm6‐YFP‐PHB. [file PBI-21-591-s003.docx]

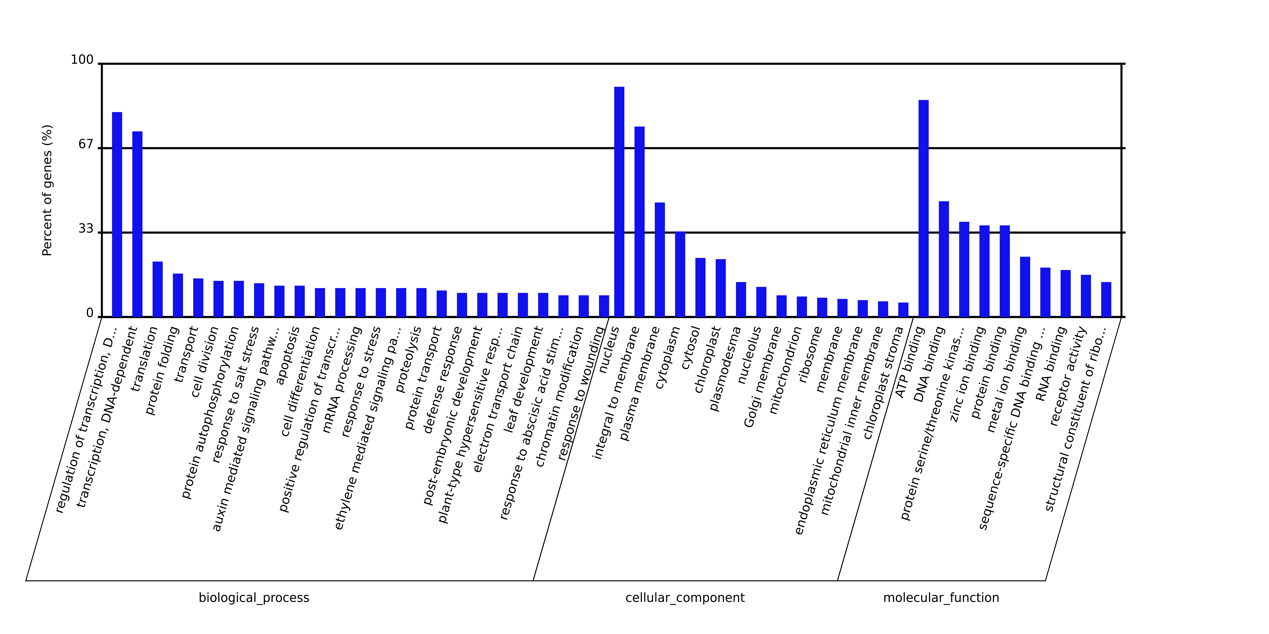


Figure S1 GO analysis of miRNA putative target genes. GO annotation categorized all of the predicted miRNA target genes and differentially expressed miRNA target genes into biological processes, cellular components, and molecular functions.


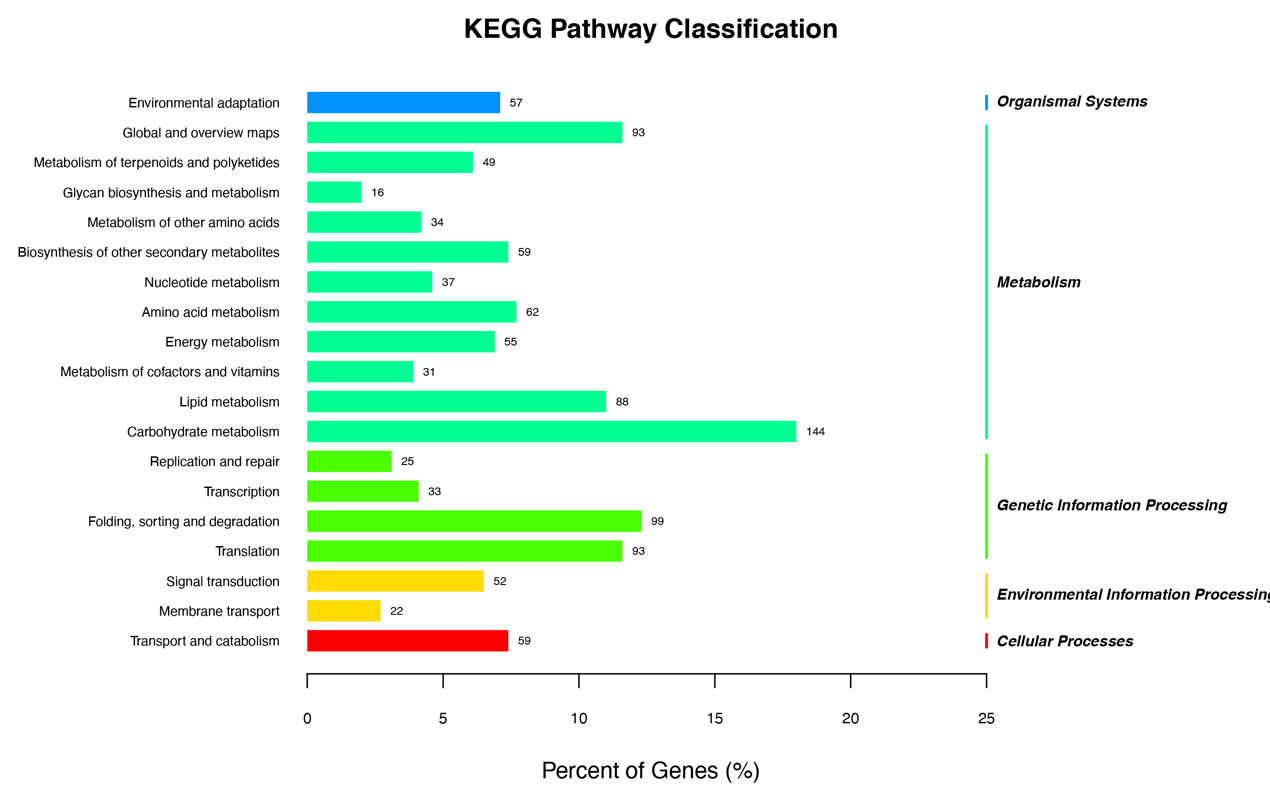


Figure S2 KEGG analysis of miRNA putative target genes. Functional annotation of KEGG pathways in *A. annua* by KEGG database.


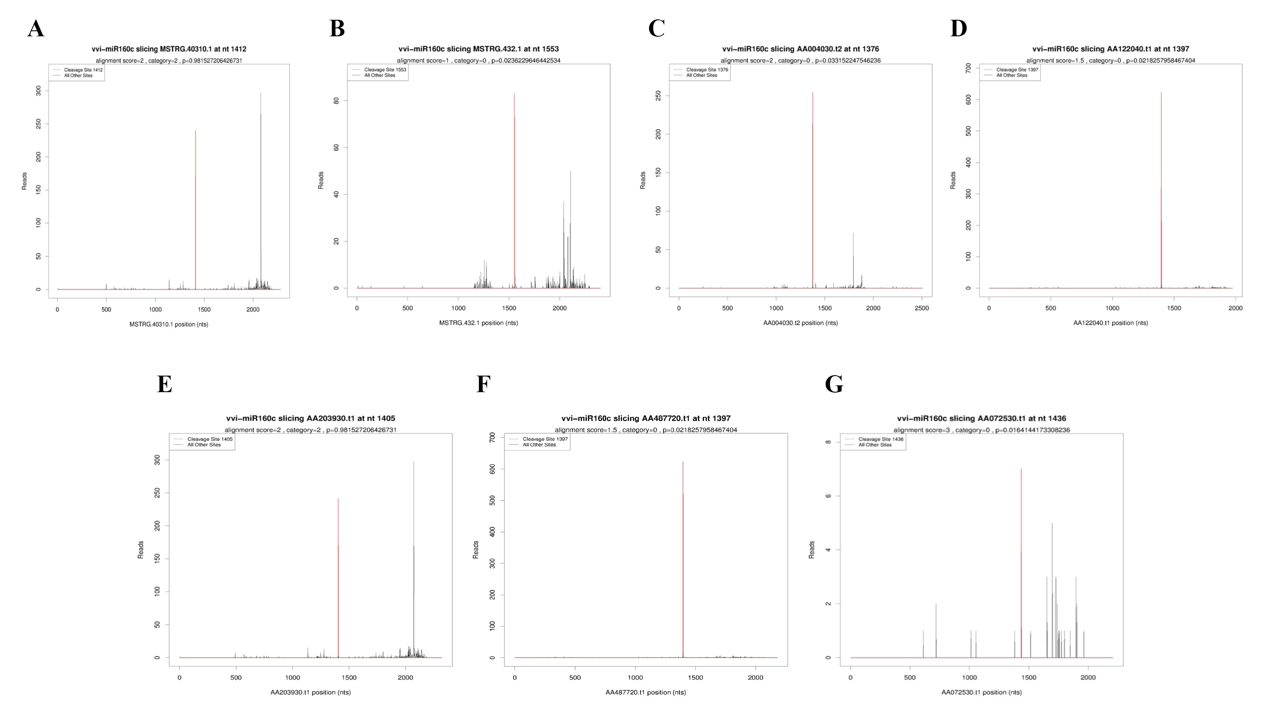


Figure S3 Target plots of the targets cleaved by the miR160. The T-plots show the distribution of the degradome tags along the full-length of the target mRNA sequence. The red line represents the predicted cleavage site of the corresponding miRNAs. (A-G) Cleavage features in *ARF1-ARF7* mRNA by miR160 from the degradome library, respectively.


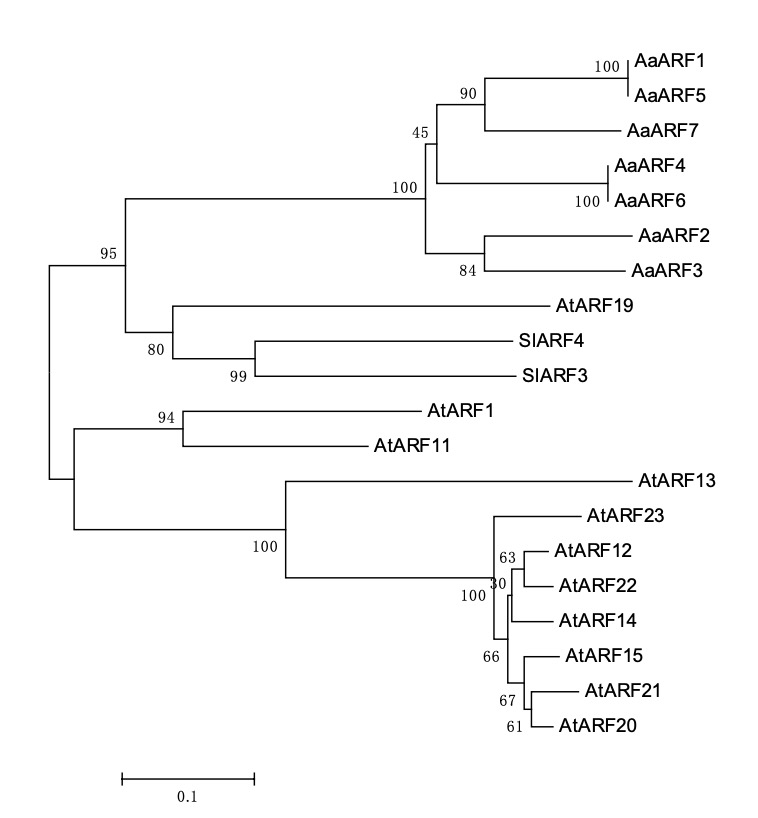


Figure S4 Neighbor-joining phylogenetic tree of ARF from *A. annua*, tomato, and *Arabidopsis*. Sequences were aligned using Clustal W and the phylogenetic tree constructed with MEGA. Bootstrap values were obtained for 1000 replications.


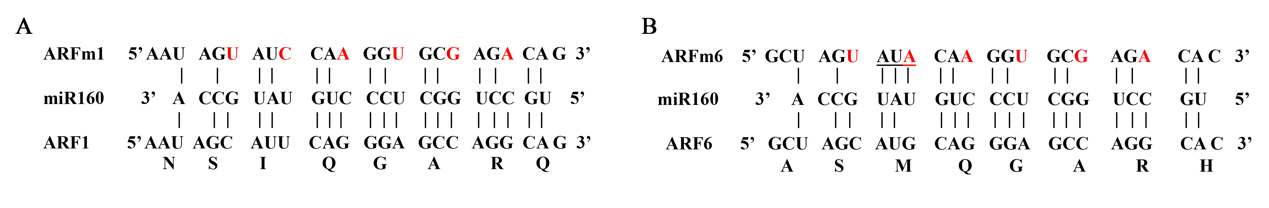


Figure S5 The mutant nucleotides in *ARFm1* and *ARFm6* with red character. Although *ARFm6* causes a Met-to-Ile amino acid substitution (underlined codon), *ARFm6* does not change the amino acid sequence.


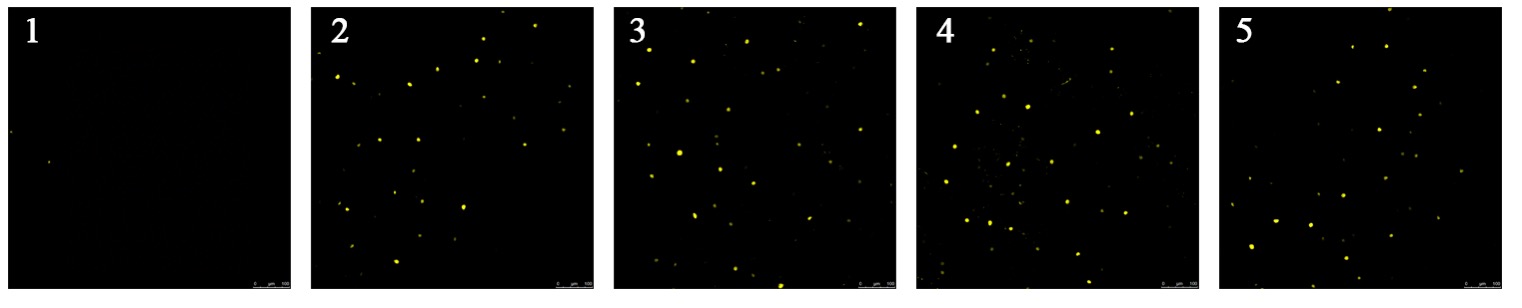


Figure S6 miR160 targeted ARF6 were verified in *N. benthamiana* leaves. 1, miR160-PHB; 2, ARF6-YFP-PHB; 3, miR160-PHB+ARF6-YFP-PHB; 4, ARFm6-YFP-PHB; 5, miR160-PHB+ARFm6-YFP-PHB.
