# Supplementary material for: Profiling of phytohormone‐specific microRNAs and characterization of the miR160‐ARF1 module involved in glandular trichome development and artemisinin biosynthesis in Artemisia annua
Source: Plant Biotechnol J. 2022 Dec 20;21(3):591–605. doi: 10.1111/pbi.13974 (PMC9946145; doi:10.1111/pbi.13974)
Supplement: Supplementary file 5 — Table S4 Stem‐loop primers of miRNA for reverse transcription, primers of miRNA for qRT‐PCR. Table S5 Primers for RLM‐5′ RACE. Table S6 Primers used for plasmid construction in this study. [file PBI-21-591-s004.docx]

Table S4 Stem-loop primers of miRNA for reverse transcription, primers of miRNA for qRT-PCR

| miR_name | RT primer (5’-3’) | Forward primer (5’-3’) |
| --- | --- | --- |
| miR160 | GTCGTATCCAGTGCAGGGTCCGAGGTATTCGCACTGGATACGACTGGCAT | AACCTTGCCTGGCTCCCTGT |
| miR156 | GTCGTATCCAGTGCAGGGTCCGAGGTATTCGCACTGGATACGACGTGCTC | CGGCGGTTGACAGAAGATAGA |
| miR408 | GTCGTATCCAGTGCAGGGTCCGAGGTATTCGCACTGGATACGACGCCAGG | TCGGCTTGCACTGCCTCTTC |
| miR171 | GTCGTATCCAGTGCAGGGTCCGAGGTATTCGCACTGGATACGACGATATT | TCGCTTGATTGAGCCGTGCC |
| miR166 | GTCGTATCCAGTGCAGGGTCCGAGGTATTCGCACTGGATACGACGCGGAG | GCCGCCGATGTTGCTTATAGGA |
| miR159 | GTCGTATCCAGTGCAGGGTCCGAGGTATTCGCACTGGATACGACTATGGT | TCGCGTACCGTCCTAGTCTCA |
| miR164 | GTCGTATCCAGTGCAGGGTCCGAGGTATTCGCACTGGATACGACAATGAC | GCGGCGGCTCTCTATGCTTCT |
| miR477 | GTCGTATCCAGTGCAGGGTCCGAGGTATTCGCACTGGATACGACTGGCAT | TTCCTTGCCTGGCTCCCTGT |
| miR390 | GTCGTATCCAGTGCAGGGTCCGAGGTATTCGCACTGGATACGACTGGCAT | TTCCTTGCCTGGCTCCCTGG |
| miR6114 | GTCGTATCCAGTGCAGGGTCCGAGGTATTCGCACTGGATACGACTATGCT | CGGCGGTGACAGAAGAGAGTG |
| Universal reverse primer | | GTGCAGGGTCCGAGGT |
| Actin-F | | CCAGGCTGTTCAGTCTCTGTAT |
| Actin-R | | CGCTCGGTAAGGATCTTCATCA |
| qPCR-ARF1-F | | TGGCGGACACTAACACAGAT |
| qPCR-ARF1-R | | CCGCCGTTATTTGCATCAGA |

Table S5 Primers for RLM-5*'* RACE

| Primer | Sequence 5*'*-3*'* |
| --- | --- |
| ARF1-outer | AACCCGCCGCTAAACTCCTA |
| ARF1-inner | GCCTTTGTGCCGTCTTTGAA |
| ARF2-outer | ACGCATCCAATGTCCAGTCA |
| ARF2-inner | TGAAGGACATGGGACTGCAG |
| ARF3-outer | CTGCAACGGCATAGTTGCTT |
| ARF3-inner | GCCAATATCCTCAGACTCCAGA |
| ARF4-outer | CACTGCTCGAATCCGTTAGA |
| ARF4-inner | CCGGTCACGTCCCAATAATG |
| ARF5-outer | GAATCTGAACTAGCGACGACTG |
| ARF5-inner | CCAGTTGAGGTCCGGTAAAT |
| ARF6-outer | GCCAGAGTTGCTAGTCTCATTC |
| ARF6-inner | GCCAACGTCTTCTGATTCCATG |
| ARF7-outer | CCCACAACCTCCATAACAAAGC |
| ARF7-inner | TCACTAAAGGGTTCATCTCC |
| 5*'* RACE Outer Primer | GCTGATGGCGATGAATGAACACTG |
| 5*'* RACE Inner Primer | CGCGGATCCGAACACTGCGTTTGCTGGCTTTGATG |

Table S6 Primers used for plasmid construction in this study

| Primer | Sequence(5’-3’) | Experiment |
| --- | --- | --- |
| miR160-F | GGAGGGTGAAGGAATCAACA | Gene clone |
| miR160-R | CCGCTACCCTTCAATTAACC |  |
| ARF1-F | GTTCTGTTGTTGTATGCCATCGGC |  |
| ARF1-R | GCAAGAATCTGAACTAGCGACGAC |  |
| ARF6-F | TGCAAGAAGGCAATGAGAGTCTGC |  |
| ARF6-R | CCAAACTGATCGATTGCTGCTG |  |
| miR160-PHB-F | TCTCTCTCTAAGCTT**GGATCC**CACCCACTACCATCACACTC | Overexpression vector construction |
| miR160-PHB-R | CTTATCGATACCGTC**ACTAGT**TTACACCGCTACCCTTCAAT |  |
| STTM160-PHB-F | TCTCTCTCTAAGCTT**GGATC**CTGGCATACAGGCTAGAGCCA |  |
| STTM160-PHB-R | CTTATCGATACCGTC**ACTAGT**TGCCTGGCTCTAGCCTGTATGCCAATTCTTCTTCTT |  |
| PHB-ARF1-F | TCTCTCTCTAAGCTT**GGATCC**ATGATTAATGTAATGGATCA |  |
| PHB-ARF1-R | CTTATCGATACCGTC**ACTAGT**AACTCCTACATTGTCGCTCC |  |
| PHB-ARFm1-F | **TAGTATCCAAGGTGCGAGACA**TAAT  CCGCAATTCGGATTTGATCATCA |  |
| PHB-ARFm1-R | **TGTCTCGCACCTTGGATACTA**TTGG  GAATGTGATTGGAGATCATAGAT |  |
| RNAi-ARF1-F | AAATCTAGAAAACCATGGCATCTTTCACCATTTTCGCC | RNAi vector construction |
| RNAi-ARF1-R | AAAGGATCCAAAGGTACCTTGTTCAGTAAAAATTGGTTG |  |
| M13F | GTAAAACGACGGCCAGT | PCR identification |
| M13R | CAGGAAACAGCTATGAC |  |
| hpt-F | CGATTTGTGTACGCCCGACAGTC |  |
| hpt-R | CGATGTAGGAGGGCGTGGATATG |  |
| JDPDK-F | ACAGTGGTCCCAAAGATGGA |  |
| JDPDK-R | GGCGGTAAGGATCTGAGCTA |  |
| JDPDK-1F | TTGGATTGATTACAGTTGGGA |  |
| JDPDK-1R | CTTCTTCGTCTTACACATCAC |  |
| rbcsr | ATTAACTTCGGTCATTAGAGGC |  |
| YFP-ARF1-F | ATGGACGAGCTGTACAAGGGATCCATGATTAATGTAATGGATCA | Transient expression |
| YFP-ARF1-R | CTAGAGGATCAATTCGAGCTCAACTCCTACATTGTCGCTCC |  |
| YFP-ARF6-F | ATGGACGAGCTGTACAAGGGATCCATGATTACTTTTATGGATTC |  |
| YFP-ARF6-R | CTAGAGGATCAATTCGAGCTCAGAATCTACATTGTTTTCAC |  |
| YFP-ARFm1-F | TAGTATCCAAGGTGCGAGACATAATCCGCAATTCGGATTTGATCATCA |  |
| YFP-ARFm1-R | TGTCTCGCACCTTGGATACTATTGGGAATGTGATTGGAGATCATAGAT |  |
| YFP-ARFm6-F | TAGTATACAAGGTGCGAGACACACACGTTACGGTTTATCTTTATCAGA |  |
| YFP-ARFm6-R | TGTCTCGCACCTTGTATACTAGCAGGATGATGATGAAGGTTCAAATTC |  |
| pB42AD-ARF1-F | GATTATGCCTCTCCCGAATTCATGATTAATGTAATGGATCATCATATGAA | Y1H |
| pB42AD-ARF1-R | AGAAGTCCAAAGCTTCTCGAGCTAAACTCCTACATTGTCGCTCC |  |
| pDBR2-GAGACA-BOX2-F | AATTCTATGTTCGAGACACGGTGGCTATGTTCGAGACACGGTGGCC |  |
| pDBR2-GAGACA-BOX2-R | TCGAGGCCACCGTGTCTCGAACATAGCCACCGTGTCTCGAACATAG |  |
| ARF1-pGEX-F | GATCTGGTTCCGCGTGGATCCATGATTAATGTAATGGATCATCATATGAA | EMSA |
| ARF1-pGEX-R | TCAGTCAGTCACGATGCGGCCGCCTAAACTCCTACATTGTCGCTCCC |  |
| pGreen0800-DBR2-F | CGGTATCGATAAGCTTAAGAACTTCGAGATAGAAAA | Dual-LUC |
| pGreen0800-DBR2-R | ATCCCCCGGGCTGCAGTCAGTGATGGAGTTGGTAAA |  |
